# A framework for doctoral education in developing students’ mental well-being by integrating the demand and resources of the program: An integrative review

**DOI:** 10.12688/f1000research.131766.1

**Published:** 2023-04-21

**Authors:** Vrinda Acharya, Ambigai Rajendran, Sandeep Shenoy

**Affiliations:** 1 Department of Commerce, Manipal Academy of Higher Education, Manipal, Karnataka, 576104, India

**Keywords:** Challenge-hindrance demands, well-being, doctoral students, Job Demands-Resources model, doctoral education

## Abstract

**Background:**Research on doctoral students’ mental well-being has gained significant importance in recent years. The findings of such studies were uncertain about the critical demands and resources of a doctoral program that substantially influence the students’ mental health. This review aims to integrate the current evidence in bringing out the nature and significance of differentiated demands, contextual and personal resources, and their influence on the well-being of the students.

**Methods:** An integrative literature review was conducted based on the five-stage framework of Whittemore and Knafl. The study identified 45 articles published from 2000 onwards following the Joanna Briggs Institute quality evaluation criteria and PRISMA reporting guidelines for selecting eligible articles.

**Results:** The integrative review findings divulge that differentiated demands of doctoral programs were categorized into challenge-hindrance demands. The differentiated demands experienced by doctoral students were grouped as ambiguity in doctoral program structure, resource inadequacy, workload, complexity, and responsibility. Additionally, institutional support, research supervisory support, and intrinsic motivation were treated as essential resource in mitigating the effects of the differentiated demands of the doctoral program.

**Conclusions:** An integrated conceptual model was built exclusively for doctoral programs and suggests that the universities and supervisors design and structure healthy, constructive doctoral programs. As an outcome of the review, theoretical underpinnings of demands-resources and mental well-being are reported. The current review is an initial attempt to synthesize challenge-hindrance demands and contextual-personal resources in determining the mental well-being of doctoral students.

## 1. Introduction

Growing mental health problems are the major contributor to the global health burden; hence there is a need to address this concern in various settings. Mental health refers to individuals’ social, psychological, and emotional well-being. Ensuring the good psychological health of an individual improves their potential to work and productivity. Several studies witnessed the mental health problems of doctoral students and are at high risk compared to the general population, i.e., 32% of the students experience psychological disorders because of stress, depression, and anxiety ( Darley, 2021; Herrmann & Wichmann-Hansen, 2017; Levecque *et al.*, 2017). Psychological distress among doctoral students results in program dropout, reduced quality research outputs, academic disengagement, detachment from learning and development activities, and work-family conflict.

Evidence has confirmed the rise in mental health problems among doctoral students were viz: rigorous research policies, dynamic work conditions with increased program demands, a growing number of publication targets, financial commitments to manage the research and family, conflicting inputs from the expert panel, resulted in unpleasant experience in maintaining work-life balance of the doctoral students ( Levecque *et al.*, 2017; Pappa *et al.*, 2020; Skopek *et al.*, 2020). These existing studies have highlighted the various stressors of doctoral students subjectively instead of demarcating the intensity of the stressors as challenge-hindrance demands. A recent study by McCauley and Hinojosa (2020) conceptualized doctoral demands as challenge-hindrance stressors that influence the learning experience of doctoral students. The study claims that hindrance demands strain doctoral students and challenge demands motivate them in professional development. However, the effect of these demands on mental health of the student in the presence of resources remains unanswered. Therefore, studies that examine the degree of challenge-hindrance demands in the context of doctoral students are missing.

Job Demands-Resources (JD-R) model postulates that high demands and inadequate resources result in growing academic strain ( Pappa *et al.*, 2020) and distress among doctoral students ( Sufyan & Ali Ghouri, 2020). Mental disorder caused by the lack of resources that includes: less scope for receiving research grants ( Maloshonok & Terentev, 2019), inadequate training from institutions and supervisor, lack of research infrastructure at institutions, limited emotional-technical support and guidance from the supervisor, insufficient access to peer and family support and financial responsibilities ( Curtin *et al.*, 2016; Haven *et al.*, 2019; McCauley & Hinojosa, 2020). The resources from institutions, supervisors, family, and peers are likely to moderate doctoral students’ stress and are considered contextual resources ( Cornwall *et al.*, 2019; Lynch *et al.*, 2018). At the same time, studies investigated the explicit requirement of intrinsic motivation rather than any other personal resources ( Matheka *et al.*, 2020; Ryan *et al.*, 2021; Sverdlik & Hall, 2019). However, the moderating role of contextual and personal resources in the presence of high demands has not been applied to doctoral education in JD-R model.

Prior studies on doctoral program challenge and their impact on students’ well-being are often inconclusive to build a comprehensive model. Thus, we propose a framework, by identifying demands-resources of doctoral education, using what we learn from academic literature. Existing literature offers rich sources of input in developing a current integrated framework and offers a quality insight for doctoral educators, policymakers, institutions, and supervisors.

## 2. Theoretical background

Studies have adopted Bronfenbrenner’s ecological framework by considering the personal, supervisor, university, family, and community as multi-faceted factors influencing doctoral students’ mental health status ( Beasy *et al.*, 2019; Usher & McCormack, 2021). As anticipated by Bronfenbrenner’s ecological framework, also grounded on the JD-R model, this section explores the antecedents of mental well-being at supervisor, university, and doctoral students’ levels.

### 2.1 The Job Demands-Resources model

JD-R model is a leading stress-coping model that explains the well-being of an employee ( Demerouti *et al.*, 2001). This model highlights that the demands and resources stem from the job, and its imbalance causes burnout and ill-being. Although the JD-R model is designed to study employees’ well-being in the work environment, it has also been applied to the learning environment. In the doctoral education context, job demands refer to the demands appraised by the researchers in their doctoral studies and roles that may influence their mental health ( Levecque *et al.*, 2017). Job resources refer to “support from the institution, supervisors, peers, and family that may foster students’ motivation and reduce health impairment by buffering against the negative impact of job demands”. Doctoral programs’ demands and resources are originated from various sources during the program journey, that influence the students’ well-being ( Pyhältö *et al.*, 2012). These research demands and resources are appraised differently by doctoral researchers, institutions, and supervisors ( Ellis *et al.*, 2015). Prior studies used the JD-R model to explain the intensified strain in the doctoral journey is due to high program demands and limited resources. However, these studies fail to predict its impact on mental health of the students ( Haven *et al.*, 2019; Ryan *et al.*, 2021; Sufyan & Ali Ghouri, 2020). Therefore, we reviewed all the prior studies to understand the doctoral programs’ demands and resources that influence doctoral students’ well-being at three levels (i.e., institutions, supervisor and personal).

### 2.2 Differentiated demands

Individuals appraise a stressful situation or work environment either as a challenge or hindrance demands. These demands are conceptualized as “two factor stress model” or ‘differentiated demands model’ ( Cavanaugh *et al.*, 2000; LePine *et al.*, 2005). Challenge demands promote the desirable outcomes such as learning, job satisfaction, and organizational commitment. In contrast, hindrance demands lead to destructive outcomes, such as withdrawal behavior, burnout, and turnover intentions ( LePine *et al.*, 2005). Individuals experience burnout due to hindrance demands, whereas challenge demands can be overcome by acquiring an essential resource ( Ellis *et al.*, 2015). Hindrance demands include role conflict, organizational politics, role ambiguity, administrative hassles, and interpersonal conflict. Challenge demands include time pressure, workload, and responsibility ( Lepine *et al.*, 2005). This dual-process framework of job demands has been examined in the working environment, but limited studies are reported in the doctoral education environment ( McCauley & Hinojosa, 2020). In summary, it is vital to examine the influence of dual role of doctoral program demands of the doctoral journey that impacts the mental health of doctoral students.

### 2.3 Doctoral Program’s contextual resources

Job resources positively affect a worker’s accomplishment, learning, growth, and psychological and physical well-being ( Demerouti *et al.*, 2001). The Conservation of Resources (COR) theory separates job resources into contextual and personal resources. Contextual resources are those located outside the individual and are related to work or social environment ( Hobfoll, 2001). In doctoral education, contextual resources include supervisory support, the social environment in which the doctoral student is situated, and the institution’s research training ( Sufyan & Ali Ghouri, 2020). During doctoral studies, contextual resources may be obtained from outside (e.g., from family or friends) or within the institution (e.g., from the institution, supervisor, or peers) ( Skopek *et al.*, 2020; Waight & Giordano, 2018). A study by Dericks *et al.* (2019) empirically analyzed the combination of peer, supervisor, and department support as a contextual resource in predicting doctoral students’ satisfaction. Critical resources from the institutions and transparency in the program requirements enhanced the Ph.D. completion rates ( Skopek *et al.*, 2020). Despite the moderating role of contextual resources in the stressful environment, there is a dearth of studies that underpin its influence on doctoral students’ mental health.

### 2.4 Personal resources

Personal resources refer to individual characteristics that contribute to goal achievements by lessening the negative influence of job demands and positively influencing their well-being ( Ellis *et al.*, 2015; Hobfoll, 2001). Significant personal resources include self-efficacy, optimism, intrinsic motivation, and resilience ( Schaufeli & Taris, 2014). Personal resources of the doctoral student are more critical than contextual resources in fostering program completion ( Pyhältö *et al.*, 2012). Many theories reflect the robustness of personal resources in the doctoral journey. One such theory is Self-Determination Theory (SDT), conceptualized based on intrinsic motivation, which is critical personal resource of the doctoral students. Intrinsic motivation refers to doctoral students’ innate interest, enjoyment, and excitement in pursuing a Ph.D. ( Litalien & Guay, 2015). A student with high intrinsic motivation devotes time for research, and contributing to knowledge ( Shin *et al.*, 2018). Existing studies have reported a strong link between intrinsic motivation and doctoral students’ well-being ( De Clercq *et al.*, 2021). However, there is lag of studies on doctoral programs that insight the moderating role of intrinsic motivation between the mental health well-being and demands.

### 2.5 Mental well-being of doctoral students

The well-being of a doctoral student refers to the state of mind of the researcher that is primarily influenced by the demands of their role and the support provided by the program ( Juniper *et al.*, 2012). The empirical findings confirm a positive association between employees’ mental well-being and favorable individual and organizational-level outcomes ( Sverdlik & Hall, 2019). The previous studies have examined various antecedents of the well-being of doctoral students, including domain-specific expertise, inadequate social support, unclear expectations from supervisors, access to financial resources, relationships mismatch with supervisors, and the scholarly community networks ( Barry *et al.*, 2018; Juniper *et al.*, 2012; Marais *et al.*, 2018).

The previous literature has enumerated different factors of mental well-being of doctoral students, but there is limited evidence that theorized the distinct nature of dual role of demands and contextual-personal resources. There is a lack of clarity in the past studies on how the demands of doctoral programs are categorised in determining its positive and negative impact on well-being. Also there exists a paucity of the literature that examines the multiple and inter-connected resources that enhance the well-being of the students. The doctoral education is inextricably associated with a stressful journey, and we suggest that the intrinsic motivation of the student moderates the program’s high demands on the mental health of the students. However, little is known about the significance of intrinsic motivation in the framework of JD-R model.

To address the above-mentioned gap in the literature, the present study adopted the integrative review method. We adopted Bronfenbrenner’s ecological framework, which recognizes that multiple and inter-connected demands and resources impact students’ well-being status. Thus, this study classifies the cause of the mental health problems among doctoral students as either challenge or hindrance demands, and we illustrate the utility of the contextual-personal resources in shaping the well-being of the doctoral students. The current review offers opportunities for university advisors and institutions to comprehend and design the policy structure of doctoral education fully. We propose theory-driven recommendations for doctoral educators to maximize the challenge demands of the program rather than a hindrance and offer supportive contextual resources in the program journey.

## 3. Methodology

According to Whittemore and Knafl (2005) and Hopia *et al.* (2016), the “integrative review method helps the reviewer to assess the methodological clarity, compare the data, analyze and generate the patterns within the selected articles” (pp. 550–551). Integrative reviews are performed to synthesize the previously published theoretical and empirical research as policy initiation. This methodology focuses on understanding the broad constructs, relationships, and theory-driven reasoning to design a framework for the proposed study ( Torraco, 2005). The review has adopted five steps of integrative review methodology suggested by Whittemore and Knafl (2005): (1) problem formulation and setting of the broad purpose and review questions, (2) literature search, (3) quality and relevance appraisal of selected literature, (4) data analysis through data abstraction, comparison, and synthesis of the selected article, and (5) the presentation of results. The reviewers have been involved in the Joanna Briggs Institute (JBI) standard for quality evaluation and interpretation of the selected articles ( The Joanna Briggs Institute, 2016). After conducting the preliminary search, the reviewers performed title and abstract screening to check the articles as per the eligibility criteria by the first two authors to exclude irrelevant reports. Articles rejected by both authors were not included in the review during the title and abstract screening process. In abstract screening, approved abstracts by either author are included in the review. Then the final search of full-text screening is performed for reports accepted by both authors. Suppose any difference of opinion among authors on the inclusion criteria was resolved by discussion in the presence of a third author and reached a consensus. The included studies were saved in M.S. excel, and duplicates were removed. Subsequently, the selected studies were classified based on the methodology of the studies.

### 3.1 Problem formulation

Compared to the general population, doctoral students are at increased risk of experiencing stress and mental health problems, including anxiety and depression ( Hazell *et al.*, 2020; Levecque *et al.*, 2017). The existing studies on doctoral education recommended the integration of JD-R and differentiated demands for assessing the mental well-being of doctoral students. Three review questions were framed by integrating the JD-R and differentiated demands. (1) What are the challenge-hindrance demands of the doctoral program that impact the mental health of doctoral students? (2) What contextual and personal resources moderate doctoral students’ stress? (3) What relevant guiding theories strengthen doctoral students’ well-being? These questions aided in synthesizing our research results, which enabled us to review the prior work and formulate an integrated framework for doctoral education. The proposed framework can be validated further by a quantitative approach.

### 3.2 Search method

The literature search was conducted using two databases, namely Scopus, and Web of Science, using the Boolean operators as keywords. The following were the keywords used as search strategy: “doctoral program” OR “doctoral education” OR “doctoral scholar” OR “doctoral student” AND “differentiated demands” AND “challenge and hindrance”, AND “JD-R” AND “motivation” AND “well-being” AND “mental health” AND “stressors”. A similar Boolean operation was done for the “doctoral candidates”, “Ph.D. scholar” and “Ph.D. students”. The author performed the manual search by reviewing the cited articles from the selected list to include the relevant articles.

*3.2.1 Inclusion and exclusion criteria*

**Figure 1.**
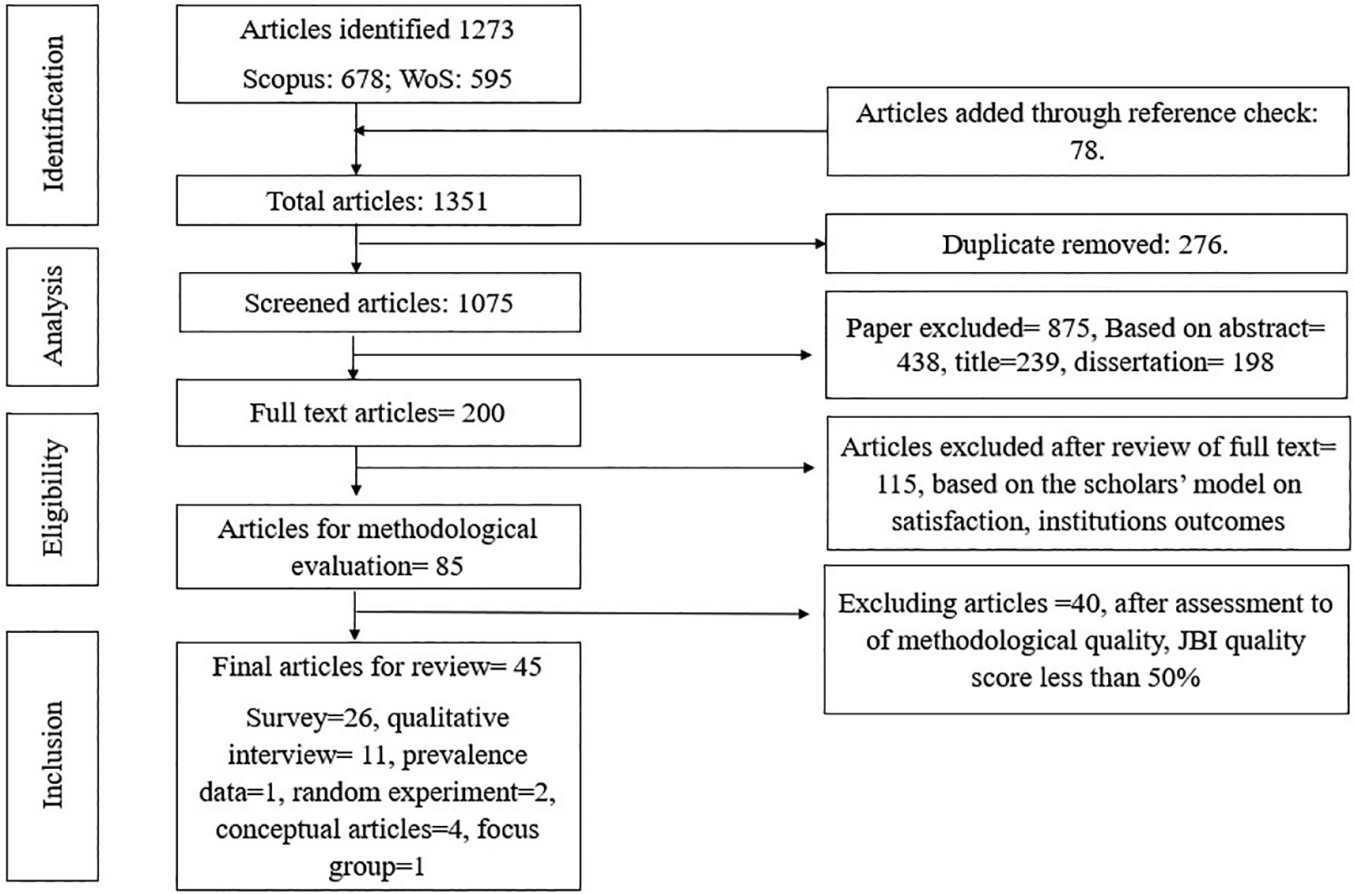
Flow chart of the integrative review selection process.

Studies undertaken in an “academic setting” were considered for inclusion, which had been determined by the criteria that include studies on the JD-R model, motivation studies, differentiated job demands, and well-being among doctoral students. Also, quantitative, qualitative, conceptual, and mixed methods studies published in the English language from 2000 onwards were included because of the significant transformation in structured doctoral education across different countries starting from the 20th century onward ( Kot & Hendel, 2012). The reviewers excluded the studies beyond the scope of the above inclusion search criteria. The articles focusing on institutional or individual outcomes were excluded from the study, for example, program satisfaction, Ph.D. dropout rate, and students’ performance. Based on the quality scores allotted by the reviewers as per JBI, the articles not meeting the quality requirement were excluded. The articles were chosen based on whether the selected final articles had been empirically tested, and theoretically supported. Finally, 45 studies were included in the review (see Figure 1). We followed the PRISMA integrative review checklist to identify, select, appraise, and synthesise studies.

### 3.3 Critical quality appraisal

Quality appraisal of the selected articles was performed using the JBI quality appraisal checklist. This tool estimates the article’s methodological quality, the possibility of bias in the design, analysis, reliability, and validity measure used in the selected article. Two review authors independently assessed the selected articles’ quality assessment. The third author helped to resolve discrepancies that arose during the process. This method helped to overcome the bias during the assessment phase. In Annexures A to E in the extended data ( Acharya *et al.,* 2023a), a collated quality assessment checklist is given for reference. Based on JBI critical appraisal checklist, we have exacted a total of 26 cross-sectional design studies: two articles on the randomized experiment, one observational study, four conceptual papers, and 12 qualitative studies for the data analysis.

### 3.4 Data analysis and presentation

The selected articles were screened based on methodological process, antecedents, consequences, theoretical articulation, and facilitators of mental well-being of doctoral students. From the final 45 articles, the associated variables were identified, iteratively compared, coded, categorized, and summarized for an integrated conclusion (see Table 1).

**Table 1.** Summary of included studies.

Author, year, country	Research design, Method (sample size of doctoral students)	Theory	Key finding/outcome
Van Rooij *et al.* (2021), Netherlands	Quantitative cross-sectional survey (n=839)	Basic Psychological Needs of SDT	Quality of the supervisor-scholar relationship, doctoral student’s perception of belonging, the level of autonomy was positively associated with satisfaction and inversely affected the program leave intentions.
Devenish *et al.* (2009), Australia	Qualitative semi-structured interview (n=12)	Conventional economic theory	Peer to informal peer support is the most valuable enabler for doctoral progress by mutual empowering.
Waight & Giordano (2018), UK	Qualitative semi-structured interview (n=35)	Nil	Several doctoral students turn into external non-academic support as online resources, family, and personal doctor rather than institutional support.
Dericks *et al.* (2019), UK	Quantitative cross-sectional survey (n=409)	Nil	A significant predictor of doctoral students’ satisfaction is supervisor support. Also, the support from department and academic qualities influences the doctoral students’ satisfaction.
Caesens *et al.* (2014), Belgium	Quantitative cross-sectional survey (n=194)	COR	Organization and supervisor support with job satisfaction and perceived stress is mediated by engagement. Supervisor support is the powerful support and co-worker support benefits in informal mentoring.
Devos *et al.* (2015), Belgium	Qualitative semi-structured interview (n=21)	SDT	There presents a thin borderline between control and structure supervisor support also with autonomy and controlling. A high level of autonomy support from supervisor is perceived as positive by the doctoral students.
Skopek *et al.* (2020)	Prevalence study (n=30)	Nil	The strength of supervision and support is essential for program structure by providing clear deadlines and sufficient funding extension.
Curtin *et al.* (2016), USA	Quantitative cross-sectional survey (n=1173)	SCT	All three mentoring (instrumental, sponsorship, and psychosocial) predict the feelings of self-efficacy; pursue an academic career, interest in a career the goal.
Sin *et al.* (2021)	Focus groups (n=30)	Nil	Students believe that compulsory coursework does not add value to their training; instead, its relevance increases when they choose courses related to their research topic, and the selected courses are practical orientation.
Guerin *et al.* (2015), Australia	Quantitative cross-sectional survey (n=405)	Nil	Ph.D. student success is resulted from five factors: Intrinsic motivation, career progression, family and friends, supervisor influence, study involvement
Matheka *et al.* (2020), Kenya	Quantitative cross-sectional survey (n=628)	Expectancy-value theory	Ph.D. students’ success is not significantly influenced by extrinsic motivation, but it is positively influenced by intrinsic motivation. Self-efficacy negatively predicts students’ success.
Litalien *et al.* (2015), Canada and the US	Quantitative cross-sectional survey (n=244, n=1060)	SDT	motivational scale with second order constructs: intrinsic, integrated, identified, introjected, and external, motivation is a major predictor of the doctoral degree completion.
Litalien and Guay (2015), Canada, US	Quantitative cross-sectional survey (n=244, n=1060)	SDT	perceived support by advisor and by faculty have positive relationship with completion and dropout intentions, through autonomous regulation
Lynch *et al.* (2018), Russia	Quantitative cross-sectional survey (n=112)	SDT	A close personal relationship supports all basic psychological needs. Academic supports the doctoral students’ autonomy and competence, in comparison to supervisor support.
Marais *et al.* (2018), French	Randomized controlled trial (n=846)	SDT	Mental health problems among doctoral students are due to a deficiency in study involvement during thesis writing and career training. Intervention effect in reduction of anxiety has been testified in a study using control and test groups.
Sverdlik and Hall (2019), USA	Quantitative cross-sectional survey (n=3004)	SDT	During the coursework phase, doctoral students reported internally motivated and highest well-being, while this score is lowest during the comprehensive examination phase.
McAlpine *et al.* (2022), Swiss	Quantitative cross-sectional survey (n=123)	Nil	Positive life-work relations diminish the risk of exhaustion, leading to cynicism and burnout among doctoral students
Barry *et al.* (2018), Australia	Qualitative semi-structured interview (n=81)	Nil	Doctoral students reported advanced levels of stress, depression, anxiety than the general population.
Herrmann and Wichmann (2017), Danish	Quantitative cross-sectional survey (n=1670)	Nil	QPPQ scale has good psychometric properties with the constructs as a collegial research environment, harsh tone, insecurity, loneliness, exhaustion, and ownership.
Haven *et al.* (2019)	Quantitative cross-sectional survey (n=129)	Nil	PPQr is validated and reliable scale to evaluate academic publication pressure in all domains. The scores are intensely related to emotional exhaustion scores.
Pappa *et al.* (2020), Finnish	Qualitative semi-structured interview (n=5)	Nil	The study stressors were challenges in the research journey, intrapersonal regulation, funding of the doctoral study, career prospects, and lack of a supportive network. Personal resources act as a motivational force and mediate in mitigating the stress.
Maloshonok and Terentev (2019), Russia	Qualitative semi-structured interview (n=11)	Nil	hurdles in the completing the doctoral educations are: Structural of education, heterogeneous program goals, universities are not prepared for the massive expansion, excessive dependence on the supervisor, lack of writing skills for publications.
Cornwall *et al.* (2019), New Zealand	Qualitative semi-structured interview (n=152)	Cognitive stress theory	The study reported that the stressors were uncertain about doctoral education structure, financial burdens, time pressure, and adjustment in scholarly communities.
Janssen *et al.* (2021), Netherland	Qualitative semi-structured interview (n=18)	SDT	Doctoral student and supervisor misalignment is observed in all three types of basic needs that leading to tension. Need-based schemas help in establishing the influential association between supervisor-scholar.
Sufyan and Ali Ghouri (2020), UK	Conceptual paper	Demand-Resource, (COR)	The Demand-Resource model and COR deliver mechanisms to identify stress either as routine or stressful situations. A peer support model to prevent and mitigate the stress by emotional, instrumental, informational, and social companionship.
Levecque *et al.* (2017), Belgium	Quantitative cross-sectional survey (n=3659)	Nil	One-third of doctoral students are at risk of having depression, higher than the general population, students, and employees’ groups. Organizational policies, the work-family interface, job control, the leadership style of supervisor, autonomy to make decisions are influence the frequency of mental distress among the doctoral students.
Juniper *et al.* (2012), UK	Quantitative cross-sectional survey (n=2500)	Nil	The well-being scale of doctoral students reported the acceptable reliability and validity with seven constructs: Home and health, research, supervisor, development, facilities, social, and university.
Stubb *et al.* (2011), Finland	Quantitative cross-sectional survey (n=669)	Nil	More than half of students reported a significant source of burden is the scholarly community. Feelings of empowerment positively influence study engagement.
McCauley and Hinojosa. (2020).	Conceptual paper	Nil	The individual doctoral students appraise hindrance or challenging stressors as a response to stress they have experienced.
Beasy *et al.* (2019)	Quantitative cross-sectional survey (n=222)	social cognitive career theory	Doctoral students reported minimal supports. Their experiences are captured through Bronfenbrenner’s bioecological systems model to understand the performative practices in research.
Usher and McCormack (2021	Quantitative cross-sectional survey (n=532)	Bourdieu’s social reproduction theory	Age, gender, nationality, work status, and program years significantly impact students’ experiences, motivation, self-confidence, and mental well-being.
Pyhältö *et al.* (2012)	Quantitative cross-sectional survey (n=669)	JD-R model	Resources and challenges perceived by the doctoral students and supervisor linked to the doctoral students’ study satisfaction and supervisory relationship.
Ellis *et al.* (2015)	Conceptual paper	JD-R, the transactional theory of stress	The study identified the individual and work-related factors of the stress of newcomers and resources to cope with the program's demands.
Pervez *et al*. (2021)	Quantitative cross-sectional survey (n=113)	Nil	Doctoral students experience significant anxiety, depression, and impostor syndrome than the general population. Supervisor and peer support are negatively related to depression and anxiety.
Ryan *et al.* (2021)	Quantitative cross-sectional survey (n=595)	JD-R model	The major four themes were support services, peer engagement and networking, culture and community, supervisors, and supervision practices.
Kemp *et al.* (2014)	Qualitative semi-structured interview (n=20)	SDT	Motivational orientation such as external and introjected strongly associated with isolation, disengagement, and poor learning outcomes.
Cornér, and Pyhältö (2019)	Qualitative semi-structured interview (n=15)	JD-R model	The supervisor reported a higher level of challenges than the available resources. Challenges include structural elements of the research community, whereas resources are a social aspect of the work and individual competence.
Byrom *et al.* (2022)	Quantitative cross-sectional survey (n=431)	Nil	Supervisory relationships, good general health, family support, sleep, and low levels of self-depreciation reported by the doctoral student robust mental well-being and it lower the stress levels.
De Clercq *et al.* (2021)	Quantitative cross-sectional survey (n=461)	SDT	Five types of motivation were identified corresponding to different combinations of satisfaction of doctoral students’ psychological needs.
Hazell *et al.* (2020)	Conceptual paper	Nil	DRs reported greater levels of stress than the general population. The review reported the heterogeneous and disparate consequences of doctoral students' mental health risks, such as isolation and protective factors, including social support.
Crook *et al.* (2021)	Quantitative cross-sectional survey (n=585)	Nil	PGRs reported lower well-being and higher depression, anxiety compared to the general population. Well-being was positively correlated with personal and professional relationships and negatively with academic challenges and mental health problems.
Boone *et al.* (2020)	Qualitative semi-structured interview- (n=49)	Theory of persistence	Doctoral students are motivated by family, friends' support, and religious beliefs. Faculty members motivate doctoral students through individual coaching, faculty-student relationships, providing university resources, and clarifying program requirements.
Barry *et al.* (2019)	Randomized controlled trial (n=34)	Nil	The doctoral students in intervention group reported a statistically significant reduction of depression and increased self-efficacy, hope, and resilience compared to the control group.
Mason (2012)	Quantitative cross-sectional survey (n=125)	SDT	A positive relationship is significant with motivation and psychological needs. Autonomy and relatedness need mediate the doctoral students' study satisfaction.
Kulikowski and Potoczek (2019)	Quantitative cross-sectional survey (n=360)	JD-R model	Seven primary resources most strongly related to Ph.D. student satisfaction.

*3.4.1.1 Differentiated demands and doctoral students’ well-being*

Doctoral students are required to work for long hours, present the findings of their work regularly, learn rigorous methodologies and analysis skills, obtain tangible research outcomes in terms of high-quality publications, and balance the student-supervisor relationship ( Litalien & Guay, 2015; Sufyan & Ali Ghouri, 2020). To meet these bounded requirements, complexity, study responsibility and workload of the doctoral program have increased and are considered as challenge demands ( Pervez *et al.*, 2021). The doctoral program also confined with a lack of transparency in the program, communication gap between the supervisor-student, low quality mandatory coursework, lack of infrastructures, which hinder the progress of doctoral studies. ( Levecque *et al.*, 2017; Sin *et al.*, 2021). These hindrance demands summarize the ambiguity in the doctoral program, poor relationship with the supervisor, family, and advisory members, and resource inadequacy that threatens the students’ mental health ( McCauley & Hinojosa, 2020). The students appraise challenge demands of the doctoral program as an opportunity to advance and hindrance demands as a threat to their learning ( McCauley & Hinojosa, 2020). In summary, challenge demands positively influence the well-being of the student, and hindrance demands adversely influence.

*3.4.1.2 Contextual resources of doctoral programs*

Job resources support doctoral students in achieving their goals, research growth, and development. A recent integrative review of the JD-R model postulated the significance of resources at three levels: the institution level, the team level (supervisor style and co-workers), and the individual level (personal resources) ( Kwon & Kim, 2020). Similarly, empirical works on doctoral programs examined the primary resources from the institution, supervisor, and personnel is essential in maintaining doctoral students’ well-being ( Ryan *et al.*, 2021; Waight & Giordano, 2018). Access to research learning infrastructure, financial assistance in the form of scholarship, transparency in the policy structure, and valuable guidance from the scholarly community are referred to as contextual resources provided by the institutions that enhance the intrinsic motivation of doctoral students ( Janssen *et al.*, 2021). Emotional support from family, online mentoring groups, face-to-face support from peers and faculty members, are the social resources that assist the doctoral student in dealing with stress ( Boone *et al.*, 2020). Supervisors’ open and honest communication, emotional and technical support, flexible supervision, patience, insightfulness, and the propensity to honour the research students’ self-reflection and listening skills are significant supervisor resources that facilitate the students’ well-being ( Dericks *et al.*, 2019). Contextual resources such as supervisors and social support buffer the negative relationship between hindrance demands and work engagement. Job resources also boost the positive association of challenge demands and work engagement ( Tadić *et al.*, 2015). 

*3.4.1.3 Intrinsic motivation as a personal resource in the doctoral study*

Unlike other personal resources, the intrinsic motivation is a significant internal resource of doctoral students that has been witnessed in literature. Intrinsic motivation is the internal desire to study, that is critical in achieving program completion ( Dericks *et al.*, 2019; Matheka *et al.*, 2020). Intrinsic motivation of the doctoral student is often associated with their positive personal experience and its influence on improving their performance and academic engagement ( Litalien & Guay, 2015). Intrinsic motivation limits the influence of hindrance demands on the strain and, in turn, improves the mental health of the doctoral student ( McCauley & Hinojosa, 2020). Intrinsically motivated doctoral students undertake doctoral education due to their inherent interest in acquiring knowledge in their domain area and are capable of withstanding hindrance demands ( Matheka *et al.*, 2020). Intrinsic motivation with contextual resources supports doctoral learning outcomes and reduces the effect of burnout ( van den Broeck *et al.*, 2011). Empirical studies have highlighted the moderating role of intrinsic motivation in strengthening job resources and basic psychological needs ( van den Broeck *et al.*, 2011).

*3.4.1.4 Significance of well-being in JD-R model*

Existing evidence recommend that adoption of JD-R model in doctoral education setting would allow to examine the influence of challenges on two different levels of outcomes i.e., organisation and individual level. ( McAlpine *et al.*, 2022). Also, studies to date have focused mainly on mental health problems or mental distress rather than the positive side of the construct. Therefore, the current study sheds light on the mental well-being of doctoral students by considering both positive and negative perspectives of doctoral program demands and resources.

*3.4.2 Guiding and contributing theories*

In explaining the challenge-hindrance demands, the current review evolved from Folkman and Lazarus’s (1985) Transactional Model of Stress and Coping (TMSC). The theory argues that doctoral student appraises the program demands depending on their subjective environment as challenge or hindrance demands. The theory also contends that hindrance demands can be a source of stress, and challenge demands support the personal resources to lessen the individual strain ( Pervez *et al.*, 2021). A limited number of studies analyzed the challenge-hindrance demands of a doctoral program that are theoretically articulated based on the TMSC model ( McCauley & Hinojosa, 2020). Nevertheless, this study did not report the influence of differentiated program demands on the well-being of students. In this connection, one may concur that there is a need for an empirical study on the magnitude of doctoral students’ appraisal of their program demands.

Using insights from the Conservation of Resources (COR) Theory, we explain the doctoral program is bound with resources along with the demands. Indeed, from the COR theory, doctoral students are always required and willing to develop the resources to manage stressful demands. Absence of these resources creates a stressful situation for the student and results in burnout. Adequate contextual and personal resources help students to mitigate work demands and maintain individual well-being. Under the COR theory, institution, supervisor, and social support are viewed as contextual resources, and intrinsic motivation is a personal resource that improves the mental health of doctoral students ( Sufyan & Ali Ghouri, 2020). Drawing on COR theory, resources gained from peer support, supervisor mentoring, learning support from institutions, and emotional support from the family will enhance an individual’s intrinsic motivation by protecting against resource loss ( Boone *et al.*, 2020). Sufyan and Ali Ghouri (2020) adopted the COR theory and JD-R model to study doctoral students’ stress. The study results conveyed that high demand and lack of resources will impact their academic journey. Thus, their findings suggest that the scope for inclusion of COR and JD-R has a significant role in determining the mental health of students.

Intrinsic motivation derives its theoretical underpinning from Causality Orientations Theory (COT), a sub-theory under SDT, stating how individuals acquaint themselves with autonomous, controlled, and impersonal external environments. COT theory drives that doctoral students to act out of their desire during the autonomous orientation. In contrast, in controlled orientation, students focuse on their gain and rewards, and they experience anxiety in impersonal orientation ( Deci *et al.*, 2001). COT suggests that the higher the degree of self-determination and self-esteem among doctoral students, the greater the autonomous orientation and the intrinsic motivation that reduce burnout levels, helping them to achieve a higher level of engagement ( Lynch *et al.*, 2018). The contrary is expected when controlled, and impersonal orientations are more predominant among doctoral students ( Litalien & Guay, 2015). The intrinsic motivation fostered by the intervention or motivational program that facilitates the researcher’s autonomous orientation instead of promoting the controlled orientation, such as reward policy, schemes, and programs.

**Figure 2.**
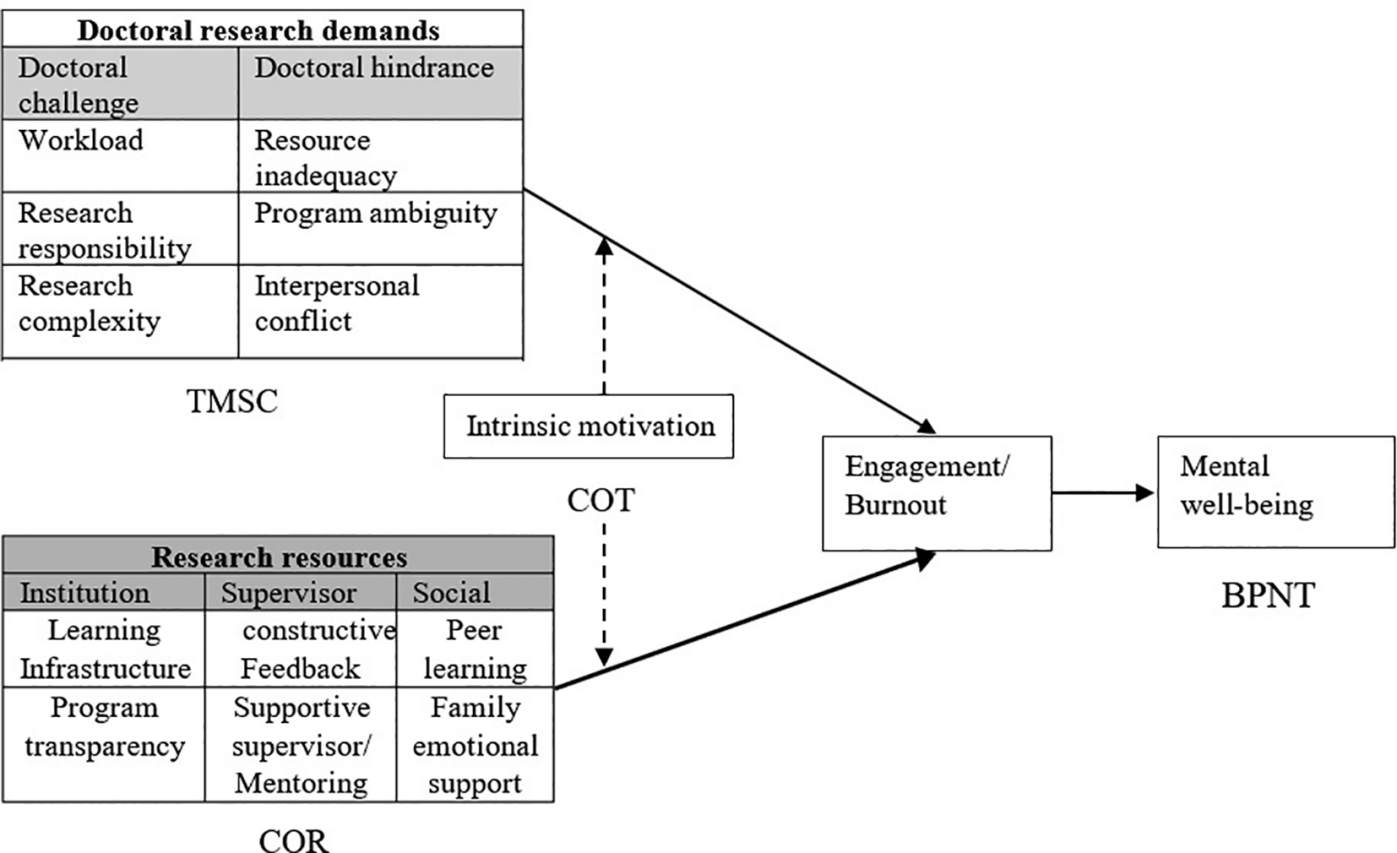
A nomological network of mental well-being of doctoral students. TMSC=Transactional model of stress and coping; COR=Conservation of Resources; COT=Causality orientations theory; BPNT=Basic Psychological Needs Theory.

The mental well-being of the present framework is underpinned by Basic Psychological Need Theory (BPNT), a mini theory under SDT that makes it novel in doctoral program research. BPNT suggests that individuals experience mental health and higher quality behavior when their social environment supports (contextual resources) their basic psychological needs ( Deci *et al.*, 2001). The social settings that boost individual well-being are termed autonomy-supportive resources ( Ryan & Deci, 2000). Autonomy-supportive resources include supervisors, faculty members, and peers that increase the doctoral students’ basic psychological needs ( Devos *et al.*, 2015; Sverdlik & Hall, 2019). In line with the BPNT, it is believed that the autonomy-supportive doctoral program setting strengthens doctoral students’ autonomy orientation ( Van Rooij *et al.*, 2021). Hence, doctoral students’ work autonomy and challenge demands induce the mental health of the researcher during the doctoral program journey. The findings of differentiated demands, resources, and mental well-being are presented in Figure 2.

## 4. Discussion

Much of the existing literature on doctoral education addresses students’ stress and mental health problems ( Barry *et al.*, 2018; Darley, 2021; Levecque *et al.*, 2017; Marais *et al.*, 2018). It is evident from the review that the existing studies need a precise classification of the doctoral program demands, resources and their interaction effect on the students’ well-being. This section discusses possible research opportunities under the theoretical purview and presents propositions that may encourage future research.

*Concept 1*

As highlighted, doctoral students undergo a tremendous amount of stress due to intrapersonal regulation, lack of supervisors’ support, completion timeline ( Cornwall *et al.*, 2019), publication targets ( Haven *et al.*, 2019), and work-life conflict ( Sufyan & Ali Ghouri, 2020). Although a significant number of articles iterates that job demands are the major stress-causing factors, surprisingly, only a few studies found the differentiating nature of job demands in the doctoral program setting ( McCauley & Hinojosa, 2020). Thus, based on the transactional theory of stress, the current review has clarified doctoral students’ challenge-hindrance demands that impact their mental health through the job strain as a mediator.

Hence operationalization of challenge and hindrance demands is proposed as follows: 1) challenge demands of doctoral programs, motivate, and inspire doctoral students to engage in research to produce high-quality research output. Moreover, 2) hindrance demands act as a constraint and impede the quality of research output. As per the proposed definition, there are two sub-categories of challenge demands: doctoral research complexity and workload demands. The categories of hindrance demands are research ambiguity demands and resource inadequacies demands. The operationalization of the challenge and hindrance demands in doctoral education are postulate as below:

Proposition 1a:
*Higher ambiguity in the doctoral program structure and insufficient resources in the program journey situate more stressful for the doctoral students, which hinders their well-being.*

Proposition 1b:
*Greater complexity and responsibility in the doctoral program foster the study engagement of the doctoral students that internally lessens the burnout.*

*Concept 2*

The current review considered Bronfenbrenner’s ecological framework to acknowledge the multi-faceted resources required for doctoral programs. With this framework, the current reviewers proposed that contextual (supervisor, institution, family) and personal (intrinsic motivation) resources, boost the doctoral students’ well-being. Waight & Giordano (2018) has highlighted the external resources in the non-academic context as online support, family, and mentor. Pervez *et al.* (2021) reported that support from supervisor and peers are essential in diminishing the impact of depression and anxiety among students. The study by Cassens *et al.* (2014) reported that support from supervisors, institutions, and co-workers mediate the relationship between perceived stress and engagement. It is surprising to see existing literature on doctoral programs independently emphasizing contextual and personal resources. Thus, the current review attempts to understand the combined effect of contextual and personal resources that enhance students’ well-being. The study has reviewed the role of supportive resources as a moderator between high demands and well-being of the student. With this, we propose the following research proposition by considering the contextual resource of doctoral programs:

Proposition 2:
*Sufficient supervisor, institutional and social resources during the doctoral journey diminish the stressful demands by protecting against future resource loss and enhancing the doctoral students’ well-being.*

*Concept 3*

Much of the existing literature on doctoral programs covered the positive influence of intrinsic motivation on students’ mental health, satisfaction, and productivity ( Kemp *et al.*, 2014; De Clercq *et al.*, 2021). Based on SDT articulation, Litalien & Guay (2015) empirically validated that autonomous orientation among doctoral students positively predict the program satisfaction, well-being, performance and is negatively associated with anxiety, turnover intention, and health impairment. Despite the 11 studies borrowed from the SDT to understand the significance of intrinsic motivation, there are only two studies have witnessed the autonomous orientation as an independent variable in predicting the well-being of doctoral students (Litalien & Guay; Litalien *et al.,* 2015). However, the literature ignored the moderating effect of intrinsic motivation in the presence of high doctoral demands. Thus, future research can also explore how the autonomous orientation moderates the influence of stress on doctoral students’ well-being. Hence, we posit the following third proposition with the significance of intrinsic motivation:

Proposition 3:
*A sense of higher autonomy emphasizes greater intrinsic motivation and lessens burnout among doctoral students. A greater level of doctoral students’ self-determination helps to achieve higher engagement that foster their well-being.*

During the integrative review, we understood that most ofthe studies were conducted on doctoral education in the UK (56%), followed by the USA (34%), and only 10% are from Asia. Since the significant studies are from USA and UK, the context-specific factors could be changed. Levecque *et al.* (2017) have reported significant overlap in doctoral education characteristics in Asian countries, the UK, the USA, and the universities across the globe, such as scholarship, enrolment protocol for the doctoral program, intensive course work, supervisors’ roles, and time-to-degree, with the notable difference in terms of fees and publication requirements. The review results align with the results that it is vital to design standard instruments to validate the differentiated demands and motivational interventions to support the mental health of doctoral students is vital ( Sufyan & Ghori, 2020). The theoretical underpinning of each construct has been reported in Table 2.

**Table 2.** Extant supportive theory for the conceptual model.

Construct	Type of the theory	Citation	Factors/dimensions
Challenge-Hindrance demands	Transactional model of stress and coping (TMSC)	McCauley and Hinojosa (2020), Ellis *et al.*, (2015)	Hindrance demands: Role ambiguity, interpersonal/role conflict, organization politics. Challenge demands: workload, complexity, time emergency, responsibility
Contextual resources	Conservation of Resources (COR)	Caesens *et al.* (2014), Sufyan and Ghouri (2020)	Social, peer, organization, supervisor, co-worker support
Personal resources (Intrinsic motivation)	Causality orientations theory (COT)	Lynch *et al.* (2018), Litalien *et al.* (2015), Marais *et al.* (2018)	Controlled motivation, autonomous motivation
MWB	Basic Psychological Needs Theory (BPNT)	Van Rooij *et al.* (2021), Devos *et al.*, (2015); Sverdlik and Hall (2019)	Competence, relatedness, and autonomy

### 4.1 Scope for future

Prior studies on JD-R model have limited their analysis to explain the influence of demands and resources on organization outcomes such as service quality and organizational commitment. Also, its impact on individual outcomes includes in-role and extra-role behaviours, creativity, and job satisfaction. There are limited studies that examined the effect of demands-resources on well-being of the individuals using JD-R model. However, future research could empirically test the influence of demands-resources on the mental health of the students in a learning context. Second, future studies can explore the effect of the demands and resources on physical health in a highly demanding environment as it reported sleep disturbance, gastrointestinal problems, frequent headaches, and eyestrain among individuals. Third, future studies can develop the framework for doctoral students’ well-being by considering the job crafting technique ( Demerouti, 2014). Here researchers are “proactive crafters” of their studies by initiating the changes in demands and resources so that they can engage themselves in their study. In line with job crafting, researchers can improve their interpersonal relationships with supervisors, peers, and scholarly communities, and learn various methodologies and academic writing skills through online collaborations.

Fourth, the mental health of doctoral researchers has considered only two factors of Ryff’s six-factor psychological well-being model, i.e., autonomy and positive relationships ( Ryff, 1989). Researchers are mandated with a high degree of autonomy, aware of the core domain knowledge, and develop a research identity. Thus, future studies might consider adopting a holistic definition of Ryff’s model to explain the mental health of the researchers.

### 4.2 Implications for higher education institutions

The proposed framework on doctoral students’ mental health serves as an implication for institutions, doctoral educators, and practitioners by incorporating the challenge demands in their doctoral education. Institutions are suggested to frame the workload of the doctoral program at the beginning of the journey that a student needs to complete and inform the students about the rationale behind the rigorous journey. Here institutions are expected to be reasonable towards students while allocating the teaching workload, providing research training, engaging administrative work, number and quality of journal publications. Doctoral programs are bound with many responsibilities that ensure the students to learn varied competencies and skills requirements throughout the journey. This research complexity is addressed by providing professional development training such as advanced statistics workshops, sessions on writing for high-impact journal publications, substantial research methodologies, and dealing with the cumbersome publication process. At the beginning of the doctoral journey the institutions need to communicate precisely with the students about the accomplishment of program responsibilities that include presenting research output at the conference, managing the research fund, and personal expenses, developing required research competencies, learning, and adopting new methodologies of the research.

Doctoral educators could assist the students in delivering the resources at three levels: institutions, supervisors, and peers. Institutions could provide funding for doctoral students to participate in professional conferences, doctoral research colloquiums, and statistical analysis workshops. Offering a research infrastructure such as separate workspaces, essential research tools, software, and databases boosts the students’ morale. Interventions such as mindfulness, stress and time management workshops, and workshops that are designed to build research resilience would assist students’ emotional and psychological development. Supervisors should assist in accumulating the supporting resources by scheduling a weekly meeting with students to discuss their research progress, providing extensive, specific, and constructive feedback, and collaborating with experts in the student’s domain. Supervisors also can act as mentors by providing the student with emotional support, respecting the student’s both personal and academic commitments such as time for family, household chores, and exercise. Supervisors also can provide authorship credits to the students by involving them in other research projects, and being friendly with students helps to preserve and protect resources for the future. Sharing research ideas with peers, networking with peers from other institutions in the same domain, participating in various research groups, and socio-emotional support from the peers, motivates the student.

Institutions and supervisors are urged to promote doctoral students’ autonomous motivation to reduce mental distress. They recommend designing an intervention that creates and promotes interest for the doctoral students in their study topic. Supervisors could be trained to encourage the students’ psychological needs, which goes beyond the research project supervision.

## Conclusion

The present integrative review developed a holistic framework of doctoral students’ mental well-being by integrating the insights from literature. From a theoretical perspective, our review has revisited the JD-R model by conceptualizing the nomological framework of demands and resources of doctoral students at the individual, supervisor, and institution levels. Due to a significant increase in mental distress among doctoral students, the study has synthesized the four critical predictors: challenges, hindrances, demands, and contextual-personal resources. Finally, the findings highlighted contributing variables for improving doctoral students’ mental health and implications for practice. Future researchers shall develop new empirical insights and apply suggested theories to understand how demands and resources interact in predicting the mental health of doctoral students.

## Data Availability

No data are associated with this article. Figshare: Quality assessment checklist by Joanna Briggs Institute (JBI), for “A framework for doctoral education in developing students’ mental well-being by integrating the demand and resources of the program: An integrative review”, https://doi.org/10.6084/m9.figshare.22298995 ( Acharya *et al., *2023a). This project contains the following extended data:
- Quality assessment checklist by Joanna Briggs Institute (JBI) Data are available under the terms of the Creative Commons Zero “No rights reserved” data waiver (CC0 Public domain). Figshare: PRISMA checklist for “A framework for doctoral education in developing students’ mental well-being by integrating the demand and resources of the program: An integrative review”, https://doi.org/10.6084/m9.figshare.22300792 ( Acharya *et al., *2023b). Data are available under the terms of the Creative Commons Zero “No rights reserved” data waiver (CC0 Public domain).
