# A framework for doctoral education in developing students’ mental well-being by integrating the demand and resources of the program: An integrative review

**DOI:** 10.12688/f1000research.131766.2

**Published:** 2023-08-29

**Authors:** Vrinda Acharya, Ambigai Rajendran, Sandeep Shenoy

**Affiliations:** 1 Department of Commerce, Manipal Academy of Higher Education, Manipal, Karnataka, 576104, India

**Keywords:** Challenge-hindrance demands, well-being, doctoral students, Job Demands-Resources model, doctoral education

## Abstract

**Background:**Research on doctoral students’ mental well-being has gained significant importance in recent years. The findings of such studies were uncertain about the critical demands and resources of a doctoral program that substantially influence the students’ mental health. This review aims to integrate the current evidence in bringing out the nature and significance of differentiated demands, contextual and personal resources, and their influence on the well-being of the students.

**Methods:** An integrative literature review was conducted based on the five-stage framework of Whittemore and Knafl. The study identified 45 articles published from 2000 onwards following the Joanna Briggs Institute quality evaluation criteria and PRISMA reporting guidelines for selecting eligible articles.

**Results:** The integrative review findings divulge that differentiated demands of doctoral programs were categorized into challenge-hindrance demands. The differentiated demands experienced by doctoral students were grouped as ambiguity in doctoral program structure, resource inadequacy, workload, complexity, and responsibility. Additionally, institutional support, research supervisory support, and intrinsic motivation were treated as essential resource in mitigating the effects of the differentiated demands of the doctoral program.

**Conclusions:** An integrated conceptual model was built exclusively for doctoral programs and suggests that the universities and supervisors design and structure healthy, constructive doctoral programs. As an outcome of the review, theoretical underpinnings of demands-resources and mental well-being are reported. The current review is an initial attempt to synthesize challenge-hindrance demands and contextual-personal resources in determining the mental well-being of doctoral students.

## 1. Introduction

Growing mental health problems are a major contributor to the global health burden, thus they must be handled in various settings. Individuals’ social, psychological, and emotional well-being are referred to as their mental health. Ensuring an individual’s psychological health increases their job potential and productivity. According to studies, doctoral students are more likely to experience mental health problems than the general population, with 32% of them witnessing psychiatric disorders as a result of stress, hopelessness, and anxiety ( Darley, 2021; Herrmann & Wichmann-Hansen, 2017; Levecque *et al.*, 2017). Psychological distress outcomes among doctoral students include dropout from doctoral programs, lower-quality research outputs, academic disengagement, detachment from learning and development activities, and work-family conflict.

Robust research policies, dynamic work environments with increased program demand, an increase in publication targets, financial obligations to manage the research and family, conflicting expert panel inputs, and an imbalance between doctoral students’ work and personal lives have all been linked to an increase in mental health issues among graduate students ( Levecque *et al.*, 2017; Pappa *et al.*, 2020; Skopek *et al.*, 2020). Existing research has highlighted the multiple issues of doctoral students rather than capturing the intensity of these stressors as challenge-hindrance demand. Doctoral program demand was conceptualised by McCauley and Hinojosa (2020) as challenge-hindrance stressors that influence a doctoral students’ learning experiences. This study found that doctoral students are stressed by hindrance demand while being driven by challenge demand in professional development. The effect of challenge-hindrance demand on student mental health in the presence of resource is still unknown. As a result, no studies have been conducted to understand the different challenge-hindrance demands that influence the mental health of doctoral students.

Substantial demand and inadequate resource in doctoral program result in increased academic strain and distress among doctoral candidates, according to the assumptions of the Job Demands-Resources model ( Sufyan & Ali Ghouri, 2020). Fewer opportunities to receive research grants ( Maloshonok & Terentev, 2019), insufficient training from institutions and supervisors, a lack of research infrastructure at institutions, limited emotional-technical support and guidance from the supervisor, insufficient peer and family support, are all indications of a lack of resource during the doctoral journey ( Curtin *et al.*, 2016; Haven *et al.*, 2019; McCauley & Hinojosa, 2020). These contextual resources are considered either institutional, supervisor, family, or peer resource, which are likely to lower doctoral students’ stress ( Cornwall *et al.*, 2019; Lynch *et al.*, 2018). At the same time, studies looked into the explicit necessity of intrinsic motivation to reduce the stress among doctoral students rather than any other personal resource ( Matheka *et al.*, 2020; Ryan *et al.*, 2021; Sverdlik & Hall, 2019). However, the moderating influence of contextual and personal resource on mental health has not been applied to doctoral education using JD-R model.

In terms of constructing a comprehensive model, prior research on doctoral program demand, resource and their impact on students’ well-being was lacking. Thus, we offer a paradigm that include doctoral program differentiated demands which hinder or drive the scholar and doctoral education contextual and personal resources necessary for the doctoral journey using what we learn from academic literature. The theoretical foundation of each construct supports a built integrative framework. This model is an important source of information for doctoral educators, policymakers, institutions, and supervisors in terms of giving high-quality insights.

## 2. Theoretical background

Bronfenbrenner’s ecological paradigm has been applied in prior studies that consider the personal, supervisor, university, family, and community as multifaceted elements impacting doctoral students’ mental health ( Beasy *et al.*, 2019; Usher & McCormack, 2021). The following section investigates the antecedents of mental well-being at the supervisor, university, and student levels using Bronfenbrenner’s ecological framework which is also structured based on the JD-R model.

### 2.1 The Job Demands-Resources model

A well-known stress-coping model that accounts for employee wellbeing is the JD-R model ( Demerouti *et al.*, 2001). The model highlights that the demands and resources stem from the job, and its imbalance causes burnout and ill-being. Although the JD-R model was created to examine employees’ well-being in the workplace, it has also been used to research and learning environments. According to Levecque *et al.* (2017) in the context of doctoral education, “job demands” refers to the obligation’s researchers have to fulfill as part of their doctoral studies and jobs that could affect their mental health. The term “job resource” refers to institutional, supervisory, peer, and family support that can shield students from the detrimental effects of work demands and help them maintain their motivation. Demand and resources for doctoral programs are generated from numerous sources over the course of the program, which affects students’ well-being ( Pyhältö *et al.*, 2012). Doctoral researchers, institutions, and supervisors appraise research demand and resource differentially ( Ellis *et al.*, 2015). The JD-R model was used to explain how the doctoral journey became more difficult due to increasing program demand and low resource availability. These studies, however, fail to anticipate its impact on students’ mental health ( Haven *et al.*, 2019; Ryan *et al.*, 2021; Sufyan & Ali Ghouri, 2020). As a result, we reviewed all prior studies to better understand the demand and resource that influence doctoral students’ well-being at three levels (i.e., institutions, supervisors, and personal).

### 2.2 Differentiated doctoral program demands

Individuals appraise stressful circumstances or work environments either as challenge demand that stimulate them, or hindrance demand which threaten them. This framework is referred to “differentiated demand model” ( Cavanaugh *et al.*, 2000; LePine *et al.*, 2005). Challenge demand encourage desired outcomes, including learning, job satisfaction, and organisational commitment, whereas hindrance demand result in negative consequences such as withdrawal symptoms, burnout, and turnover intentions ( LePine *et al.*, 2005). Hindrance demand cause burnout, whereas individuals can overcome challenge demand by acquiring a necessary resource ( Ellis *et al.*, 2015). Role conflict, organisational politics, role ambiguity, administrative hassles, and interpersonal conflict are examples of hindrance demand. Time constraints, workload, and job responsibility are challenge demand ( Lepine *et al.*, 2005). Few studies have been reported on this challenge-hindrance demands framework in the doctoral education setting, compared to the working environment ( McCauley & Hinojosa, 2020). Consequently, it is essential to identify the differentiated demands that affect the mental health of doctoral students, which assist the educator restructure their program.

### 2.3 Doctoral Program’s contextual resource

According to Demerouti *et al.* (2001) job resource favorably impacts a worker’s accomplishments, learning, growth, and psychological and physical well-being. Based on the Conservation of Resources theory (COR), job resources might be contextual or personal. Contextual resources are situated outside the individual and are related to the workplace or social setting ( Hobfoll, 2001). Contextual resource for doctoral students can be outside (e.g., peers, supervisors, family, or friends) or within the university ( Skopek *et al.*, 2020; Waight & Giordano, 2018). The doctoral student’s social environment, the institution’s research training, and supervisory support are contextual resource in doctoral education ( Sufyan & Ali Ghouri, 2020). The combined effect of peer, supervisor, and department support was empirically analyzed by Dericks *et al.* (2019) as a contextual resource in predicting doctoral students’ satisfaction. The doctoral completion rates were improved by critical institutional resource and transparency in the program criteria ( Skopek *et al.*, 2020). Few studies support the impact of contextual resource on doctoral students’ mental health; however, the moderating effect they play in stressful settings is ignored.

### 2.4 Personal resources of doctoral students

Personal resource are the traits of an individual which help them to achieve their goals by reducing the adverse effects of their work demand thereby improving their well-being ( Ellis *et al.*, 2015; Hobfoll, 2001). Essential personal resource that has been researched in the past include resilience, self-efficacy, optimism, and intrinsic motivation ( Schaufeli & Taris, 2014). To encourage program completion, a doctoral student’s personal resource is more critical than contextual resource ( Pyhältö *et al.*, 2012). The strength of personal resource during the doctoral journey is reflected in many theories. Self-Determination Theory (SDT) is one such theory, which conceptualized the intrinsic motivation as crucial personal resource for doctoral candidate. Studies on doctoral education resources has highlighted the importance of cultivating intrinsic motivation in doctoral students ( De Clercq *et al.*, 2021). To pursue a doctoral study, students must have an innate curiosity, delight, and excitement ( Litalien & Guay, 2015). High intrinsic motivation encourages students to invest time in learning and study ( Shin *et al.*, 2018). Existing research has shown an important association between doctoral students’ well-being and intrinsic motivation ( De Clercq *et al.*, 2021). However, studies about doctoral programs that evaluate the moderating role of intrinsic motivation between demand and mental health well-being are missing.

### 2.5 Mental well-being of doctoral students

The well-being of a doctoral student refers to the state of mind of the researcher that is primarily influenced by the demand of their role and the resource provided by the program ( Juniper *et al.*, 2012). According to empirical research ( Sverdlik & Hall, 2019), there is a positive correlation between employees’ mental health and organisational outcomes. Previous studies have looked at various factors that can affect a doctoral student’s well-being, including their supervisors’ expectations being unclear, their lack of social support, limited access to financial resource, their relationships with them being incompatible with their supervisor, and their scholarly community networks ( Barry *et al.*, 2018; Juniper *et al.*, 2012; Marais *et al.*, 2018). However, little studies support the theory of contextual-personal resources and the dual role of doctoral program demand. The differentiated demands of doctoral programs to ascertain their favourable or unfavourable effects on well-being have yet to be made clear in previous studies. More literature is also needed to explore connected resources that improve students’ well-being. Doctoral education is inextricably associated with a stressful journey. Hence, we propose that the intrinsic drive of the student moderates the program’s high demand on the student’s mental health. The fundamental role of intrinsic motivation within the JD-R model framework is not well understood yet. To consider all the doctoral program demand and resource that impact the students’ mental well-being the current study adopted the integrative review methodology, which also fulfill the aforementioned gap in the literature. We employed Bronfenbrenner’s ecological paradigm, which considers that various interrelated demand and resource. The challenge demand has roles to play in boosting study engagement; therefore, job resource should be enabled at every step of doctoral education. Individuals with great personal resource can better handle obstacles to achieving mental well-being. A detailed analysis of the student’s mental health should make these demand and resource of the doctoral program more transparent. University advisors and institutions can build the policy framework for doctoral education with the current review findings. We present theory-driven suggestions for doctoral educators to enhance program challenges and provide helpful contextual resource throughout the program journey.

## 3. Methodology

The integrative review method helps the reviewer to assess the methodological clarity, compare the data, analyse and generate the patterns within the selected articles Whittemore and Knafl (2005) and Hopia *et al.* (2016). As a first step towards policy implementation, integrative reviews are conducted to synthesise previously published theoretical and empirical studies. To build a framework for the proposed study, this methodology focuses on understanding broad construct, relationship, and theory-driven rationale ( Torraco, 2005). Whittemore and Knafl (2005) proposed five steps of integrative review methodology: (1) problem formulation that helps in setting up the broad purpose of the study using review questions, (2) literature search, (3) quality and relevance appraisal of selected literature, (4) data analysis through data abstraction, comparison, and synthesis of the selected article, and (5) presentation of results. This strategy enables the inclusion of research conducted using a range of methodologies.The reviewers of this study adopted the Joanna Briggs Institute (JBI) standard to assess the quality and interpretation of the chosen articles ( The Joanna Briggs Institute, 2016). In the preliminary step, first two reviewers performed title and abstract screening to ensure that the articles met the eligibility criteria and to exclude any irrelevant reports. During the title and abstract screening process, we eliminated the articles which is rejected by both the reviewers. The full-text screening is then conducted as a final search for articles selection approved by both authors. Disagreements among the two reviewers on the inclusion criteria were settled through discussion in a third author’s presence and reached a consensus. Duplicate articles were eliminated, and the included studies were saved in Microsoft Excel. The final articles that were selected then categorized per their methodology.

### 3.1 Problem formulation

Doctoral students are more likely than the general population to have stress and mental health problems, such as anxiety and depression ( Hazell *et al.*, 2020; Levecque *et al.*, 2017). In order to address the mental health issues facing doctorate students, the current study structured the problem statement as a research question by taking into account all the constructs that affect the students’ mental health. The existing studies on doctoral education recommended combining differentiated demand and multifaceted resource framework along with their guiding theory while evaluating doctoral students’ mental health ( McCauley & Hinojosa, 2020). We considered the following research questions to consider all the constructs of mental well-being. (1) What challenge and hindrance demand of the doctoral degree affect doctoral students’ mental health? (2) What personal and contextual resource help doctoral students cope with stress? (3) What relevant guiding theories support the well-being of doctoral students? These questions helped to synthesize our research findings, allowing us to reflect on earlier work and create an integrated framework for doctoral education.

### 3.2 Search method

Two databases, Scopus and Web of Science, were used for the literature search, and Boolean operators were used for the keyword search. As part of the search strategy, the following keywords were used: “doctoral program” OR “doctoral education” OR “doctoral scholar” OR “doctoral student” AND “differentiated demands” OR “challenge and hindrance”, AND “JD-R” AND “motivation” AND “well-being” AND “mental health” AND “stressors”. The “doctoral candidates”, “Ph.D. scholar” and “Ph.D. students” all undertook an identical Boolean procedure. In order to incorporate the relevant papers, the author conducted a manual search by going over the cited articles from the chosen list.

*3.2.1 Inclusion and exclusion criteria*

**Figure 1.**
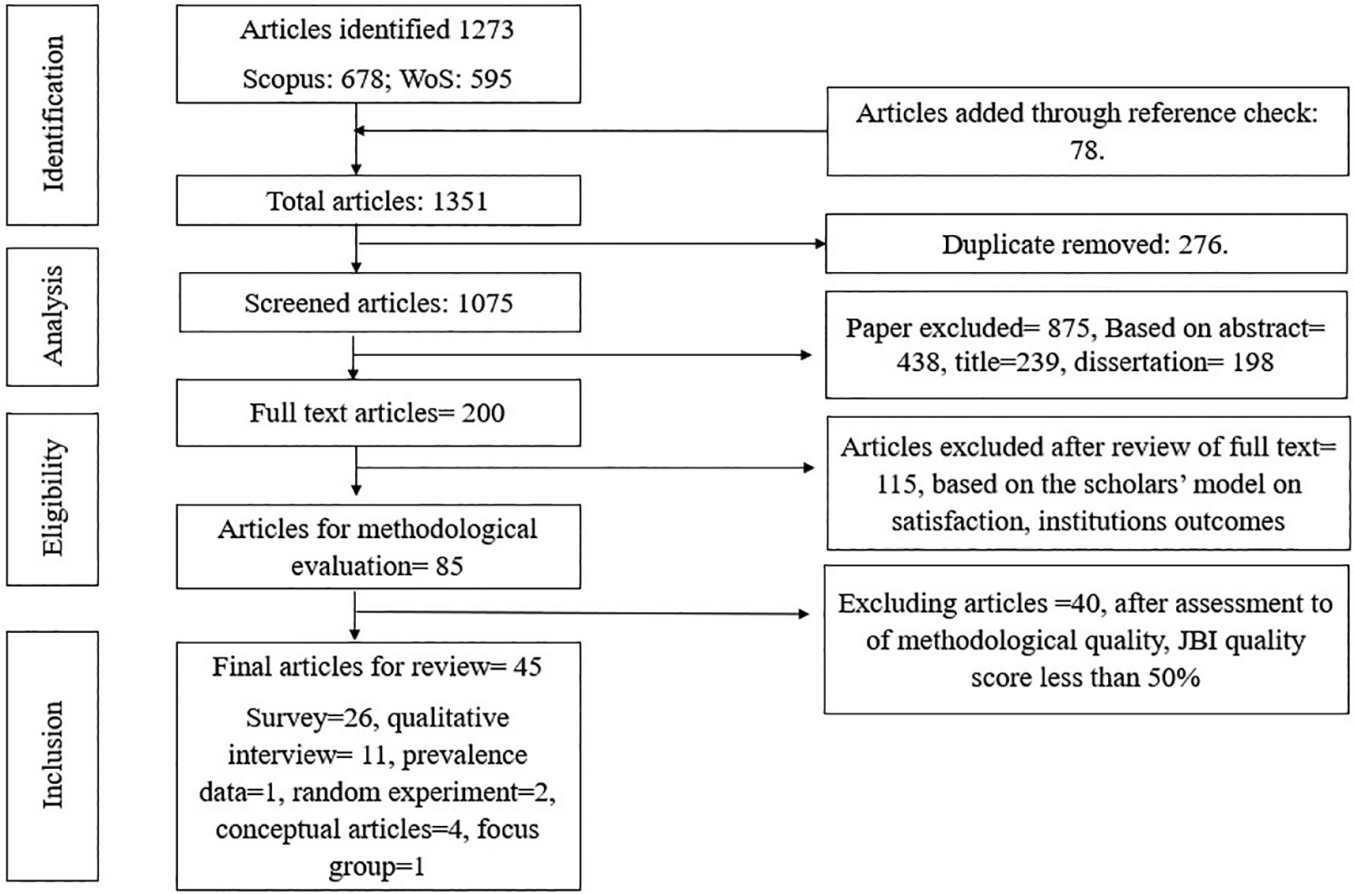
Flow chart of the integrative review selection process.

We used the PRISMA integrative review checklist to find, identify, evaluate, and synthesize studies. Studies conducted in an “academic setting”—defined by the criteria that consider research on the JD-R model, motivational studies, differentiated job demands, and well-being among doctoral students—were taken into consideration for inclusion. Due to major improvements in structured doctoral education across many nations beginning in the 20th century, the reviewer included the articles published in English from 2000 onward ( Kot & Hendel, 2012). We included the quantitative, qualitative, conceptual, review and mixed methodologies to provide a comprehensive overview of the research literature. Reviewers disqualified studies outside the purview of the aforementioned inclusion search criteria. The studies concentrating on institutional or individual outcomes, such as program satisfaction, Ph.D. dropout rate, and student performance, were eliminated from the analysis. The articles that did not match the quality standard following JBI were excluded based on the quality ratings the reviewers gave. The articles were picked depending on whether the final selections had undergone theoretical and empirical validation. Lastly, the review contained 45 papers (see Figure 1).

### 3.3 Critical quality appraisal

Using the JBI quality appraisal checklist, the selected articles’ quality was evaluated. The methodological quality of the article, the possibility of bias in the analysis, the reliability, and the validity scale employed in the selected article are all assessed using this tool. Two reviewers separately evaluated the quality of the included articles. Differences that emerged during the procedure were resolved with the assistance of the third author. This approach assisted in overcoming bias throughout the evaluation stage. The extended data ( Acharya *et al.,* 2023a), includes a quality assessment checklist that has been compiled for use in Annexures A through E. We have extracted a total of 26 cross-sectional design studies based on the JBI critical assessment checklist: two articles on the randomized experiment, one observational study, four conceptual papers, and 12 qualitative studies for the data analysis.

### 3.4 Data analysis and presentation

The selected studies were screened based on the methodological approach, causes and effects, theoretical background, and factors that support doctoral students’ mental health. The associated variables from the final 45 articles were found, and they were compared iteratively, coded, categorized, and summarised for an integrated conclusion (see Table 1).

**Table 1.** Summary of included studies.

Author, year, country	Research design, Method (sample size of doctoral students)	Theory	Key finding/outcome
Van Rooij *et al.* (2021), Netherlands	Quantitative cross-sectional survey (n=839)	Basic Psychological Needs of SDT	Quality of the supervisor-scholar relationship, doctoral student’s perception of belonging, the level of autonomy was positively associated with satisfaction and inversely affected the program leave intentions.
Devenish *et al.* (2009), Australia	Qualitative semi-structured interview (n=12)	Conventional economic theory	Peer to informal peer support is the most valuable enabler for doctoral progress by mutual empowering.
Waight & Giordano (2018), UK	Qualitative semi-structured interview (n=35)	Nil	Several doctoral students turn into external non-academic support as online resources, family, and personal doctor rather than institutional support.
Dericks *et al.* (2019), UK	Quantitative cross-sectional survey (n=409)	Nil	A significant predictor of doctoral students’ satisfaction is supervisor support. Also, the support from department and academic qualities influences the doctoral students’ satisfaction.
Caesens *et al.* (2014), Belgium	Quantitative cross-sectional survey (n=194)	COR	Organization and supervisor support with job satisfaction and perceived stress is mediated by engagement. Supervisor support is the powerful support and co-worker support benefits in informal mentoring.
Devos *et al.* (2015), Belgium	Qualitative semi-structured interview (n=21)	SDT	There presents a thin borderline between control and structure supervisor support also with autonomy and controlling. A high level of autonomy support from supervisor is perceived as positive by the doctoral students.
Skopek *et al.* (2020)	Prevalence study (n=30)	Nil	The strength of supervision and support is essential for program structure by providing clear deadlines and sufficient funding extension.
Curtin *et al.* (2016), USA	Quantitative cross-sectional survey (n=1173)	SCT	All three mentoring (instrumental, sponsorship, and psychosocial) predict the feelings of self-efficacy; pursue an academic career, interest in a career the goal.
Sin *et al.* (2021)	Focus groups (n=30)	Nil	Students believe that compulsory coursework does not add value to their training; instead, its relevance increases when they choose courses related to their research topic, and the selected courses are practical orientation.
Guerin *et al.* (2015), Australia	Quantitative cross-sectional survey (n=405)	Nil	Ph.D. student success is resulted from five factors: Intrinsic motivation, career progression, family and friends, supervisor influence, study involvement
Matheka *et al.* (2020), Kenya	Quantitative cross-sectional survey (n=628)	Expectancy-value theory	Ph.D. students’ success is not significantly influenced by extrinsic motivation, but it is positively influenced by intrinsic motivation. Self-efficacy negatively predicts students’ success.
Litalien *et al.* (2015), Canada and the US	Quantitative cross-sectional survey (n=244, n=1060)	SDT	motivational scale with second order constructs: intrinsic, integrated, identified, introjected, and external, motivation is a major predictor of the doctoral degree completion.
Litalien and Guay (2015), Canada, US	Quantitative cross-sectional survey (n=244, n=1060)	SDT	perceived support by advisor and by faculty have positive relationship with completion and dropout intentions, through autonomous regulation
Lynch *et al.* (2018), Russia	Quantitative cross-sectional survey (n=112)	SDT	A close personal relationship supports all basic psychological needs. Academic supports the doctoral students’ autonomy and competence, in comparison to supervisor support.
Marais *et al.* (2018), French	Randomized controlled trial (n=846)	SDT	Mental health problems among doctoral students are due to a deficiency in study involvement during thesis writing and career training. Intervention effect in reduction of anxiety has been testified in a study using control and test groups.
Sverdlik and Hall (2019), USA	Quantitative cross-sectional survey (n=3004)	SDT	During the coursework phase, doctoral students reported internally motivated and highest well-being, while this score is lowest during the comprehensive examination phase.
McAlpine *et al.* (2022), Swiss	Quantitative cross-sectional survey (n=123)	Nil	Positive life-work relations diminish the risk of exhaustion, leading to cynicism and burnout among doctoral students
Barry *et al.* (2018), Australia	Qualitative semi-structured interview (n=81)	Nil	Doctoral students reported advanced levels of stress, depression, anxiety than the general population.
Herrmann and Wichmann (2017), Danish	Quantitative cross-sectional survey (n=1670)	Nil	QPPQ scale has good psychometric properties with the constructs as a collegial research environment, harsh tone, insecurity, loneliness, exhaustion, and ownership.
Haven *et al.* (2019)	Quantitative cross-sectional survey (n=129)	Nil	PPQr is validated and reliable scale to evaluate academic publication pressure in all domains. The scores are intensely related to emotional exhaustion scores.
Pappa *et al.* (2020), Finnish	Qualitative semi-structured interview (n=5)	Nil	The study stressors were challenges in the research journey, intrapersonal regulation, funding of the doctoral study, career prospects, and lack of a supportive network. Personal resources act as a motivational force and mediate in mitigating the stress.
Maloshonok and Terentev (2019), Russia	Qualitative semi-structured interview (n=11)	Nil	hurdles in the completing the doctoral educations are: Structural of education, heterogeneous program goals, universities are not prepared for the massive expansion, excessive dependence on the supervisor, lack of writing skills for publications.
Cornwall *et al.* (2019), New Zealand	Qualitative semi-structured interview (n=152)	Cognitive stress theory	The study reported that the stressors were uncertain about doctoral education structure, financial burdens, time pressure, and adjustment in scholarly communities.
Janssen *et al.* (2021), Netherland	Qualitative semi-structured interview (n=18)	SDT	Doctoral student and supervisor misalignment is observed in all three types of basic needs that leading to tension. Need-based schemas help in establishing the influential association between supervisor-scholar.
Sufyan and Ali Ghouri (2020), UK	Conceptual paper	Demand-Resource, (COR)	The Demand-Resource model and COR deliver mechanisms to identify stress either as routine or stressful situations. A peer support model to prevent and mitigate the stress by emotional, instrumental, informational, and social companionship.
Levecque *et al.* (2017), Belgium	Quantitative cross-sectional survey (n=3659)	Nil	One-third of doctoral students are at risk of having depression, higher than the general population, students, and employees’ groups. Organizational policies, the work-family interface, job control, the leadership style of supervisor, autonomy to make decisions are influence the frequency of mental distress among the doctoral students.
Juniper *et al.* (2012), UK	Quantitative cross-sectional survey (n=2500)	Nil	The well-being scale of doctoral students reported the acceptable reliability and validity with seven constructs: Home and health, research, supervisor, development, facilities, social, and university.
Stubb *et al.* (2011), Finland	Quantitative cross-sectional survey (n=669)	Nil	More than half of students reported a significant source of burden is the scholarly community. Feelings of empowerment positively influence study engagement.
McCauley and Hinojosa. (2020).	Conceptual paper	Nil	The individual doctoral students appraise hindrance or challenging stressors as a response to stress they have experienced.
Beasy *et al.* (2019)	Quantitative cross-sectional survey (n=222)	social cognitive career theory	Doctoral students reported minimal supports. Their experiences are captured through Bronfenbrenner’s bioecological systems model to understand the performative practices in research.
Usher and McCormack (2021	Quantitative cross-sectional survey (n=532)	Bourdieu’s social reproduction theory	Age, gender, nationality, work status, and program years significantly impact students’ experiences, motivation, self-confidence, and mental well-being.
Pyhältö *et al.* (2012)	Quantitative cross-sectional survey (n=669)	JD-R model	Resources and challenges perceived by the doctoral students and supervisor linked to the doctoral students’ study satisfaction and supervisory relationship.
Ellis *et al.* (2015)	Conceptual paper	JD-R, the transactional theory of stress	The study identified the individual and work-related factors of the stress of newcomers and resources to cope with the program's demands.
Pervez *et al*. (2021)	Quantitative cross-sectional survey (n=113)	Nil	Doctoral students experience significant anxiety, depression, and impostor syndrome than the general population. Supervisor and peer support are negatively related to depression and anxiety.
Ryan *et al.* (2021)	Quantitative cross-sectional survey (n=595)	JD-R model	The major four themes were support services, peer engagement and networking, culture and community, supervisors, and supervision practices.
Kemp *et al.* (2014)	Qualitative semi-structured interview (n=20)	SDT	Motivational orientation such as external and introjected strongly associated with isolation, disengagement, and poor learning outcomes.
Cornér, and Pyhältö (2019)	Qualitative semi-structured interview (n=15)	JD-R model	The supervisor reported a higher level of challenges than the available resources. Challenges include structural elements of the research community, whereas resources are a social aspect of the work and individual competence.
Byrom *et al.* (2022)	Quantitative cross-sectional survey (n=431)	Nil	Supervisory relationships, good general health, family support, sleep, and low levels of self-depreciation reported by the doctoral student robust mental well-being and it lower the stress levels.
De Clercq *et al.* (2021)	Quantitative cross-sectional survey (n=461)	SDT	Five types of motivation were identified corresponding to different combinations of satisfaction of doctoral students’ psychological needs.
Hazell *et al.* (2020)	Conceptual paper	Nil	DRs reported greater levels of stress than the general population. The review reported the heterogeneous and disparate consequences of doctoral students' mental health risks, such as isolation and protective factors, including social support.
Crook *et al.* (2021)	Quantitative cross-sectional survey (n=585)	Nil	PGRs reported lower well-being and higher depression, anxiety compared to the general population. Well-being was positively correlated with personal and professional relationships and negatively with academic challenges and mental health problems.
Boone *et al.* (2020)	Qualitative semi-structured interview- (n=49)	Theory of persistence	Doctoral students are motivated by family, friends' support, and religious beliefs. Faculty members motivate doctoral students through individual coaching, faculty-student relationships, providing university resources, and clarifying program requirements.
Barry *et al.* (2019)	Randomized controlled trial (n=34)	Nil	The doctoral students in intervention group reported a statistically significant reduction of depression and increased self-efficacy, hope, and resilience compared to the control group.
Mason (2012)	Quantitative cross-sectional survey (n=125)	SDT	A positive relationship is significant with motivation and psychological needs. Autonomy and relatedness need mediate the doctoral students' study satisfaction.
Kulikowski and Potoczek (2019)	Quantitative cross-sectional survey (n=360)	JD-R model	Seven primary resources most strongly related to Ph.D. student satisfaction.

*3.4.1.1 Differentiated demands and doctoral students’ well-being*

Long hours of work, regular presentations of their research findings, learning rigorous methodologies and analysis techniques, obtaining tangible research results in the form of high-quality publications, and maintaining a healthy student-supervisor relationship are all demands placed on doctoral students ( Litalien & Guay, 2015; Sufyan & Ali Ghouri, 2020). These complexity, study responsibility, and workload of the doctoral program have grown over a period and have considered as challenge demand ( Pervez *et al.*, 2021). A lack of program clarity further constrains the doctoral program, a communication gap between the supervisor and student, low-quality required coursework, and a lack of infrastructure hinder the advancement of doctoral studies ( Levecque *et al.*, 2017; Sin *et al.*, 2021). The ambiguity in the doctoral program, the poor relationship with the supervisor, family, and advisory members, and the resource deficiency that threatens the students’ mental health are all summed up in these hindrance demand ( McCauley & Hinojosa, 2020). According to McCauley and Hinojosa (2020) student view the doctoral program’s challenge demand as a chance to advance and hindrance demand as a risk to their ability to study. In conclusion, challenge demand to benefit a student’s wellbeing, but hindrance demand negatively impact a student.

*3.4.1.2 Contextual resources of doctoral programs*

The resources of the job assist doctoral students in attaining their objectives and advancing their research. The importance of resource at three levels was hypothesized in a recent integrative review of the JD-R model: the institution level, the team level (supervisor style and co-workers), and the individual level (personal resource) ( Kwon & Kim, 2020). According to empirical studies, the contextual resources from the institution, supervisor, and social are most critical in supporting doctoral students’ well-being ( Ryan *et al.*, 2021; Waight & Giordano, 2018). Contextual resources are those offered by institution that increase the intrinsic motivation of doctoral students. These resources include access to research learning infrastructure, financial assistance through scholarship, transparency in the policy structure, and helpful advice from the scholarly community ( Janssen *et al.*, 2021). The social support helps the doctoral student to deal with stress include emotional and in-person assistance from family and peers, online mentoring groups, and more ( Boone *et al.*, 2020). Critical supervisor resource promote the well-being of the student that include open and honest communication, emotional and technical support, flexible supervision, patience level of supervisor, listening skill, and a willingness to appreciate students’ self-reflection ( Dericks *et al.*, 2019). Contextual resource like supervisors and social support mitigate the negative correlation between hindrance demand and work engagement ( Pervez *et al.*, 2021). In a non-academic setting, online support, family, and mentors are important resource ( Waight & Giordano, 2018). According to Tadić *et al.* (2015), job resource strengthens the relationship between challenge demand and job engagement. Using Bronfenbrenner’s (1979) ecological systems approach, Jackman *et al.* (2022) present interconnections between supervisors, institutions, and peers surrounding Ph.D. student for their development.

*3.4.1.3 Intrinsic motivation as a personal resource in the doctoral study*

According to studies on doctoral education, the primary personal resource needed to complete the doctoral journey is internal motivation ( Litalien & Guay, 2015). The inherent urge and drive to engage in an activity for its own sake, rather than for external incentives or demands, is referred to as intrinsic motivation (Chan *et al*., 2019). The doctoral student’s intrinsic motivation is related to their positive personal experience that enhances the doctoral student’s overall learning experience and increasing their academic engagement, performance, and chances of academic success ( Dericks *et al.*, 2019; Litalien & Guay, 2015; Matheka *et al.*, 2020). Intrinsic motivation of the doctoral student is often associated with their positive personal experience and its influence on improving their performance and academic engagement Intrinsic motivation reduces the impact of hindrance demand on occupational pressure and, as a result, enhances the mental health of doctoral students ( McCauley & Hinojosa, 2020). Intrinsically motivated doctoral student pursues doctoral education because they have an innate passion for acquiring knowledge in their domain area and can overcome hindrance ( Matheka *et al.*, 2020). Intrinsic motivation combined with contextual resource promotes doctoral learning outcomes while reducing the impact of burnout and strain ( van den Broeck *et al.*, 2011). Intrinsic motivation assists in enhancing the basic psychological needs in doctoral student ( van den Broeck *et al.*, 2011). Intrinsically motivated doctoral students feel like they can be competent to master the research challenges, autonomy to choose their interested research topic, and are part of an academic research community or group that supports their research efforts ( van den Broeck *et al.*, 2011).

*3.4.1.4 Significance of well-being in JD-R model*

Studies on doctoral well-being using the JD-R model is of utmost relevance in understanding the interaction of multiple demands and resources in enhancing doctoral students’ overall well-being ( McAlpine *et al.*, 2022). Researchers can use the JD-R model to identify specific job demands that may be detrimental to student’s mental health and design interventions to mitigate these demands (Anttila *et al*., 2021). Furthermore, rather than emphasizing the positive elements of mental health, prior studies have primarily focused on mental illnesses or distress. As a result, by considering both positive and negative elements of doctoral program demand and resources, the current study sheds light on doctoral students’ mental health. This understanding can eventually influence the development of evidence-based interventions and support systems to increase doctoral students’ well-being and achievement.

*3.4.2 Guiding and contributing theories*

In the current review the challenge-hindrance demands concept is evolved from Folkman and Lazarus’s (1985) Transactional Model of Stress and Coping (TMSC). According to the TMSC hypothesis, doctoral student appraises program demand as the challenge, or as the hindrance based on their subjective surroundings. The theory also states that challenge demand support personal resource to lower the individual strain, while hindrance demand might cause stress ( Pervez *et al.*, 2021). McCauley & Hinojosa, (2020), have examined a doctoral program’s challenge-hindrance demand impact on student’s professional development based on the TMSC model However, this study dearth in identifying the impact of various challenge hindrance program demand on students’ well-being. In this context, one would agree that an empirical study on the degree to which doctoral students appraise the demand of their program is necessary.

The doctoral study comprises resource along with demand using ideas from the Conservation of Resources (COR) Theory. Doctoral students are indeed needs to be motivated to build the resource to manage stressful expectations, according to the COR theory. The lack of these contextual resource puts student in a complex environment, leading to burnout. Student who has enough contextual and personal resource can manage their workload and retain their mental well-being. According to the COR theory, social (e.g peer and family), the institution, and the supervisor support serve as contextual resource, whereas intrinsic motivation is a personal resource that helps in preserve the doctoral students’ mental health ( Sufyan & Ali Ghouri, 2020). Employing the COR theory as a guide Boone *et al.* (2020), found that resource obtained through peer support, supervisor mentorship, learning support from institution, and emotional support from the family will increase a person’s intrinsic motivation by preventing resource loss. Sufyan and Ali Ghouri (2020) used the JD-R model and COR theory to analyze the stress experienced by doctoral student. Based on this study’s findings, their academic career will be impacted by excessive demand and a lack of resource. Thus, their findings imply that a key factor in influencing students’ mental health is the potential for inclusion of COR and JD-R.

The Causality Orientations Theory (COT), a sub-theory of SDT, which describes how people familiar with autonomous, controlled, and impersonal orientation that serves as the theoretical foundation for intrinsic motivation. Doctoral students behave in accordance with their desires during autonomous orientation, in controlled orientation, students concentrate on their benefits and rewards, while in impersonal orientation, they feel anxious ( Deci *et al.*, 2001). According to COT, higher degrees of autonomous orientation reduce burnout, allowing doctoral students to achieve higher levels of self-determination and self-esteem, in contrast, controlled or impersonal orientation ( Litalien & Guay, 2015; Lynch *et al.*, 2018). The intrinsic motivation promoted by the intervention or motivational program, such as incentive policies, schemes, and programs, supports researcher’s autonomous orientation rather than supporting the controlled orientation.

**Figure 2.**
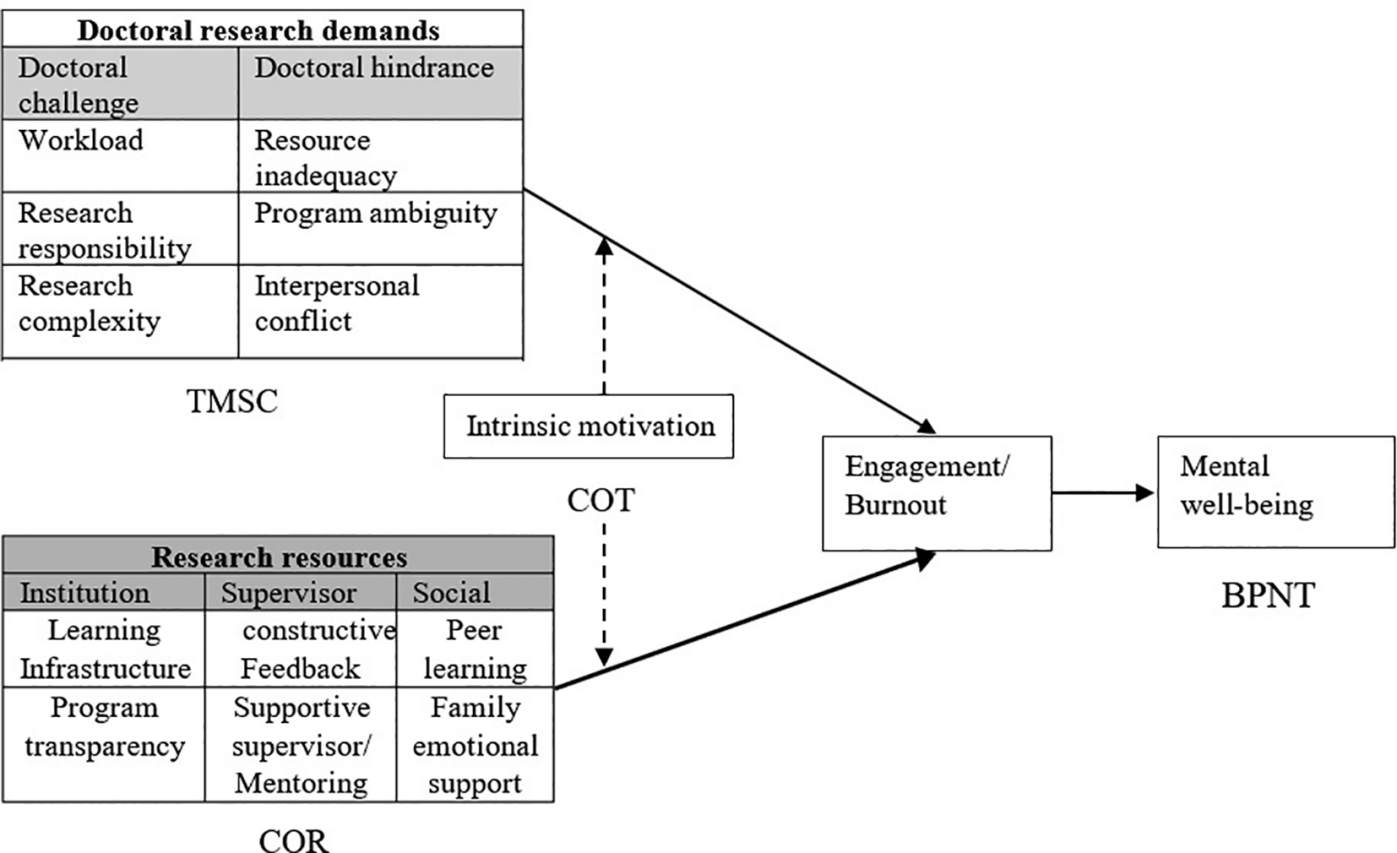
A nomological network of mental well-being of doctoral students. TMSC=Transactional model of stress and coping; COR=Conservation of Resources; COT=Causality orientations theory; BPNT=Basic Psychological Needs Theory.

The Basic Psychological Need Theory (BPNT), a mini theory under SDT, serves as the theoretical foundation for the present framework’s emphasis on mental health in doctoral program. According to BPNT, individuals exhibit better mental health and behave more ethically when their social setting support for their basic psychological needs ( Deci *et al.*, 2001). Autonomy-supportive resource are the social environments that improve individual well-being ( Ryan & Deci, 2000). Supervisors, professors, and peer are autonomy-supportive resource that help doctoral students meet their basic psychological needs ( Devos *et al.*, 2015; Sverdlik & Hall, 2019). According to the BPNT, when the doctoral program is autonomy-supportive it enhances doctoral students’ autonomy orientation ( Van Rooij *et al.*, 2021). Therefore, during the doctoral program journey, the researcher’s mental health is influenced by the work autonomy and challenge demand placed on doctoral students. Figure 2 depicts the conceptual framework for the mental well-being of the doctoral students considering the factors differential demands, resource, intrinsic motivation, and mental well-being. The theoretical underpinning of each construct has been reported in Figure 2.

## 4. Discussion

The literature on doctoral education ( Barry *et al.*, 2018; Darley, 2021; Levecque *et al.*, 2017; Marais *et al.*, 2018), addresses students’ stress and mental health problems in great detail. It is clear from the review that the current research requires a thorough description of the resource, demand, and interaction effects of this on doctoral students’ well-being. This section explores potential theoretically based study concepts and encourages ideas for potential future research through propositions.

*Concept 1*

As previously mentioned, doctoral students experience an enormous amount of stress because of intrapersonal regulations, a lack of supervisor support, program completion deadlines ( Cornwall *et al.*, 2019), publication targets ( Haven *et al.*, 2019) and problems with work-life balance ( Sufyan & Ali Ghouri, 2020). A number of articles demonstrate that job demands are substantial stressors; nevertheless, only a few studies revealed the differentiated job demands of the doctoral program setting ( McCauley & Hinojosa, 2020). The current review has therefore defined the challenge-hindrance demands that impact doctorate students’ mental health through job strain as a mediator, based on the transactional theory of stress.

Therefore, the challenge and hindrance demands are in doctoral education setting is proposed. Doctoral program demand that are stressful, however it encourages doctoral student to conduct their study and create high-quality research output referred as challenge demand of doctoral program. Furthermore, hindrance demand imposes a constraint on students and lower the quality of research output. The suggested categorization divides challenge demand into two subcategories: workload and complexity demand of doctoral research. Research ambiguity demand, and resource deficiency demand are the categories of hindrance demand. The following postulates represent the challenge and hindrance demand in doctoral education:

Proposition 1a:
Higher levels of ambiguity in the framework of a doctoral education and a lack of resources make the program journey more stressful for the doctoral students, which negatively impacts their well-being.

Proposition 1b:
The doctoral program’s increased complexity and responsibility encourage the doctoral students’ study engagement and it reduce burnout. Thus, study engagement is improved by autonomous motivation to engage in the students’ own research.

*Concept 2*

The current assessment considered Bronfenbrenner’s ecological framework to recognize the diverse resource needed for the Ph.D. program. According to this paradigm, we suggest that personal (intrinsic motivation) and environmental (supervisor, institution, family) resource enhance doctoral students’ well-being ( Pervez *et al.*, 2021; Waight & Giordano, 2018). The study by Cassens *et al.* (2014) reported that support from institutions, coworkers, and supervisors mediates the association between perceived stress and engagement. It is surprising to see a distinct emphasis on contextual and personal resource in the extant literature on doctoral programs. The current review attempts to comprehend how personal and contextual resource work together to improve students’ well-being. Considering this, and taking into account the contextual resource of doctoral programs, we suggest the following research proposition:

Proposition 2:
Sufficient supervisory, institutional, and social resource during the doctoral journey reduce stressful demand by protecting against future resource loss and improving the well-being of Ph.D. students.

*Concept 3*

The favorable effects of intrinsic motivation on students’ mental health, satisfaction, and productivity were extensively discussed in the doctoral program literature ( Kemp *et al.*, 2014; De Clercq *et al.*, 2021). According to empirical studies supported by SDT principles, Litalien and Guay (2015) found that doctoral students’ autonomous orientation predicts program satisfaction, well-being, and performance favourably and has a negative association with anxiety, intention to leave the program, and health impairment. Only two studies have demonstrated autonomous orientation as an antecedent in predicting the mental well-being of doctoral student ( Litalien *et al.,* 2015). However, the literature ignored the moderating effect of intrinsic motivation in the presence of high doctoral demand. Thus, future research can also explore how autonomous orientation moderates the influence of stress on doctoral students’ well-being. Considering the importance of intrinsic motivation, we, therefore, propose the third proposition:

Proposition 3:
Having greater autonomy enhances intrinsic motivation and prevents burnout in doctoral students. Students who perceive greater perception of self-determination are more likely to engage in activities that are positive for them.

We learned throughout the integrative review that most studies on doctoral education were conducted in the UK (56%), followed by the USA (34%), and only 10% were from Asia. The context-specific factors might be altered because the important research was from the UK and the US. According to Levecque *et al.* (2017) there is a great amount of similarity between the characteristics of doctoral education in Asian nations, the UK, the USA, and universities all over the world. Scholarships, doctoral program enrolment procedures, intensive coursework, supervisory responsibilities, and duration to a degree are among them, however, the fees for the program and publication requirements stand out as major differences. The review’s findings concur that it is crucial to develop standardized instruments to validate the diverse expectations and to develop motivating interventions to support doctoral students’ mental health ( Sufyan & Ali Ghori, 2020). The theoretical underpinning of each construct has been reported in Table 2.

**Table 2.** Extant supportive theory for the conceptual model.

Construct	Type of the theory	Citation	Factors/dimensions
Challenge-Hindrance demands	Transactional model of stress and coping (TMSC)	McCauley and Hinojosa (2020), Ellis *et al.* (2015)	Hindrance demands: Role ambiguity, interpersonal/role conflict, organization politics. Challenge demands: workload, complexity, time emergency, responsibility
Contextual resources	Conservation of Resources (COR)	Caesens *et al.* (2014), Sufyan and Ali Ghouri (2020)	Social, peer, organization, supervisor, co-worker support
Personal resources (Intrinsic motivation)	Causality orientations theory (COT)	Lynch *et al.* (2018), Litalien *et al.* (2015), Marais *et al.* (2018)	Controlled motivation, autonomous motivation
Mental well-being	Basic Psychological Needs Theory (BPNT)	Van Rooij *et al.* (2021), Devos *et al.* (2015), Sverdlik and Hall (2019)	Competence, relatedness, and autonomy

### 4.1 Future directions

Prior research on the JD-R model has focused on analysing how demand and resource affect organizational outcomes including service quality and organizational commitment. Additionally, it influences on individual outcomes includes job satisfaction, in-role and extra-role behaviors, and creativity. Limited studies have examined the interaction of demand and resource on individual’s well-being using JD-R model. Future studies, however, might use empirical observation to examine the impact of demand and resource on students’ mental health in a learning environment. Future research can examine the impact of resource and demand on individual physical wellness (such as sleep disruption, gastrointestinal issues, frequent headaches, and eyestrain) in a setting with high demand. Second, by considering the job crafting technique, future studies can provide the framework for doctoral students’ well-being ( Demerouti, 2014). By driving changes in the demand and resource of doctoral programs the scholar may devote to their research and are acting as “proactive crafters” of their studies. In keeping with job crafting, researchers can enhance their interpersonal connections with supervisors, peers, and scholarly communities through online collaborations. They can also master diverse methodologies and develop their academic writing abilities.

Third, just two of Ryff’s relationships ( Ryff, 1989) six psychological well-being factors—autonomy and positive relationships—have been considered when examining doctoral researchers’ mental health. Researchers typically tend to display a high degree of autonomy, awareness of the fundamentals of the topic, and the development of a research identity. Therefore, future studies might consider using a comprehensive definition of Ryff’s model to describe the researchers’ mental health.

### 4.2 Implications for higher education institutions

By adopting the proposed challenge-hindrance framework in doctoral education, institutions, doctoral educators, and practitioners can benefit while designing strategies to reduce doctoral students’ mental health. Recommendations for institutions include reframing the workload and responsibilities of the doctoral program at the start of the journey that a student must undertake and educate them about the rationale for this rigorous journey. Here, education institutions are supposed to discuss with students fairly while distributing the teaching load, offering research training, involving administrative duties, and increasing the quantity and calibre of journal publications. Doctoral program come with many obligations that ensure the students pick up different competencies and skills along the way. This research complexity is addressed by offering professional development training such as advanced statistics workshops, seminars on writing for high-impact journal publications, substantial research methodologies, and coping with the challenging academic publication process. The institutions must be transparent with the students about completing program requirements, which include presenting research output at conferences, managing the research fund along with personal expenses, developing the necessary research competencies.

Doctoral educators could assist the students by providing resources at three levels: peer, supervisor, and institution. Doctoral student may receive financial aid from their institutions to attend workshops on statistical analysis, doctoral research colloquia, and professional conferences. Institution may work on enhancing the students’ morale by providing a research infrastructure that includes private workspaces, necessary research tools, software for analysing the data, providing an opportunity for research collaboration with the similar topics, and research databases. Institution could facilitate interventions like mindfulness, research resilience, and stress-time-management workshops to enhance students’ emotional and psychological development. By arranging a weekly meeting with students to review their research progress, providing specific, and constructive feedback, and collaborating with experts in the student’s field, supervisors can help students gather the necessary supporting resource. Supervisor can also serve as mentor by offering emotional support for their students and showing respect for the student’s academic and personal responsibilities, such as family time, household work, and exercise. Supervisor can also authorship credits to their students by including them in other research initiatives or peers. Fostering positive relationships with students helps to maintain and preserve future resource. Peer resources in the form of socioemotional support, networking with peers from other universities in the same field, participation in various research groups, and sharing research ideas all inspire doctoral students which enhance their self-determination.

Promoting doctoral students’ autonomous motivation to lessen psychological distress by institutions and advisors is strongly advised. They advise creating an intervention that stimulates and nurtures the doctoral students’ interest in the subject of their research. Supervisors should receive training to support the psychological requirements of the students, which goes beyond research project supervision.

## 5. Conclusion

The current integrative review developed a holistic framework for understanding the mental well-being of doctoral student by incorporating the literature of the study construct. The developed conceptual integrative framework of demand and resource of doctoral program has extended the JD-R model at the individual, supervisor, and institution level and supported with theoretical standpoint. Since there has been a noticeable increase in mental distress among doctoral students, the study has merged the four indicators challenge-hindrance demand and contextual-personal resource. The study also highlighted practical implications for enhancing the mental health of doctoral students. Future researchers must generate new empirical insights and apply proposed theories to understand how demand and resource interact in predicting doctoral students’ mental health.

## Data availability

No data are associated with this article.

### Extended data

Figshare: Quality assessment checklist by Joanna Briggs Institute (JBI), for “A framework for doctoral education in developing students’ mental well-being by integrating the demand and resources of the program: An integrative review”, https://doi.org/10.6084/m9.figshare.22298995 ( Acharya *et al.,* 2023a).

This project contains the following extended data:

• Quality assessment checklist by Joanna Briggs Institute (JBI)

Data are available under the terms of the Creative Commons Zero “No rights reserved” data waiver (CC0 Public domain).

### Reporting guidelines

Figshare: PRISMA checklist for “A framework for doctoral education in developing students’ mental well-being by integrating the demand and resources of the program: An integrative review”, https://doi.org/10.6084/m9.figshare.22300792 ( Acharya *et al.,* 2023b).

Data are available under the terms of the Creative Commons Zero “No rights reserved” data waiver (CC0 Public domain).
