# Supplementary material for: A framework for doctoral education in developing students’ mental well-being by integrating the demand and resources of the program: An integrative review
Source: F1000Res. 2023 Aug 29;12:431. Originally published 2023 Apr 21. [Version 2] doi: 10.12688/f1000research.131766.2 (PMC12004065; doi:10.12688/f1000research.131766.2)
Supplement: Peer review history

# Reviewer report: Reviewer response for version 2

**DOI:** 10.5256/f1000research.154878.r375348

**Published:** 2025-04-16

**Authors:** Azad Ali

**Affiliations:** 1 Indiana University of Pennsylvania, Indiana, USA

This is a well written paper, the issue of developing student mental well-being in doctoral education is research worthy. I did not see grammatical errors and the sequence of sections is easy to follow. Well supported with relevant and recent (within last seven years) references and citations provided when needed. However, some improvements are suggested here in three areas:

 First, the explanation of the problem that this study is addressing can be elaborated on and provide supported evidence. I see explanation of the well being of mental health of doctoral students. However, how did this affected the students, did they completed, did it complicate their journey for their completion. You may refer to two articles with DoI Ref 1, 2 on causes that affect doctoral students.

 Second, the final outcome of the paper can be more specific, I know is the objective is to develop a “framework for doctoral education”, but I do not see in the conclusion section or the other section anything can be identified as “framework”. Having specific “framework” that matches what is listed in the objective could be convincing that the research objective is met. To be clear with the outcome of the research is more necessary for qualitative research because there is no hypothesis to accept or reject, thus clarity of outcome is important.

 Third, I see too many theories are discussed here and I call it “overloading with theories” because  I do not see how they all contributed to final outcome of the paper. It is good to include background theories, but overusing them may cause confusion.

 With all this in mind, I consider a very well written paper and I vote Yes for approving the indexing of this paper. 

 Thanks

Are the rationale for, and objectives of, the Systematic Review clearly stated?

Yes

Is the statistical analysis and its interpretation appropriate?

Not applicable

If this is a Living Systematic Review, is the ‘living’ method appropriate and is the search schedule clearly defined and justified? (‘Living Systematic Review’ or a variation of this term should be included in the title.)

Not applicable

Are sufficient details of the methods and analysis provided to allow replication by others?

No

Are the conclusions drawn adequately supported by the results presented in the review?

Yes

Reviewer Expertise:

Doctoral studies, Generative Artificial Intelligence, Web Design, Computer Programming

I confirm that I have read this submission and believe that I have an appropriate level of expertise to confirm that it is of an acceptable scientific standard.

## References

[1] : Developing an Evaluation Grid for the "Drawn Stories Technique": Exploring the Indicators of Children's Socio-Emotional Development, Anxiety, and Depression Levels. *J Pers Assess* .2025; 10.1080/00223891.2025.2488438 1-14 10.1080/00223891.2025.2488438 40227062

[2] : Deep Inferior Epigastric Perforator Flap Breast Reconstruction in Patients With or Without Previous Abdominal Surgery: A Systemic Review and Meta-analysis. *Plast Reconstr Surg Glob Open* .2025;13(4) : 10.1097/GOX.0000000000006701 e6701 10.1097/GOX.0000000000006701 40237008 PMC11999400

# Reviewer report: Reviewer response for version 2

**DOI:** 10.5256/f1000research.154878.r309751

**Published:** 2024-08-08

**Authors:** Dzintra Ilisko

**Affiliations:** 1 Daugavpils University, Daugavpils, Latvia

The issue of research is topical and appealing for the international readership. The current integrative review offers comprehensive holistic framework for understanding the mental well-being of doctoral student The developed conceptual integrative framework of has extended the JD-R model at the individual, supervisor, and institution level and is supported with theoretical standpoint. The paper has clear and comprehensive structure with well defined categories and visualization of data. The conclusions are short but are drawn adequately supported by the results presented in the review. The authors provided sufficient details of the methods and analysis of data. I suggest considering this study for indexing.

Are the rationale for, and objectives of, the Systematic Review clearly stated?

Yes

Is the statistical analysis and its interpretation appropriate?

Yes

If this is a Living Systematic Review, is the ‘living’ method appropriate and is the search schedule clearly defined and justified? (‘Living Systematic Review’ or a variation of this term should be included in the title.)

Yes

Are sufficient details of the methods and analysis provided to allow replication by others?

Yes

Are the conclusions drawn adequately supported by the results presented in the review?

Yes

Reviewer Expertise:

Inclusive education, education for sustainable development, educational research

I confirm that I have read this submission and believe that I have an appropriate level of expertise to confirm that it is of an acceptable scientific standard.

# Reviewer report: Reviewer response for version 1

**DOI:** 10.5256/f1000research.144636.r184790

**Published:** 2023-07-20

**Authors:** Rebekah Layton

**Affiliations:** 1 Office of Graduate Education, The University of North Carolina at Chapel Hill, Chapel Hill, North Carolina, USA

Overall: This is a well-designed systematic review on a timely and important topic, examining mental health and well-being of trainees in doctoral education - a topic which was previously under-studied but is beginning to accrue a robust literature to allow for more complex multi-study examinations such as the current work. The authors embed their review in sound theoretical frameworks, taking a holistic view of both mental health (negative) and well-being (positive). The authors effectively enumerate factors in doctoral training which can contribute to mental health and well-being outcomes that have been studied. Another strength of this paper is the consideration and inclusion of the work of international scholars broadly and across academic settings. Important themes, implications, and recommendations are provided. While this work has excellent merit, the writing has consistent errors that obscure the important content the authors are trying to convey. Examples and suggestions for improving readability are included below. This is the main area of improvement needed to elevate this manuscript to a high-quality publication. I hope the authors choose to revise this manuscript to increase the readability and writing quality, which is the primary barrier to it currently being indexable work.

 As this is a systematic review article, statistical analyses are not applicable; it is difficult to evaluate whether conclusions are adequately supported in some cases due to the errors in word choice and sentence structure - hence, if this can be revised to be more readable - it is likely this issue would be resolved. This has the potential to provide a valuable contribution to the literature with some additional revisions and careful editing.

 Example Edits/Wording Needed:

 1. The word "significant" is (over) used in a number of places that are confusing to the reader - especially since the authors are not referring to statistical analyses - alternative word choices should  be used whenever possible to avoid creating the impression that a statistical result is being described. The word significant/significance should be replaced throughout, to the greatest extent possible. For example:

- Abstract - "has gained significant importance in recent years"
- "little is known about the significance of intrinsic motivation..."
-Although a significant number of articles iterates..."

2. A number of verbs are used out of context to the extent that the sentence no longer makes sense; in some cases, I could guess at the meaning I thought the authors intended, but in other cases the meaning was unclear.

- Abstract - "findings divulge"
- "studies witnessed the the mental health problems of doctoral students.."
- "Although a significant number of articles iterates..." - maybe consider: "a number of articles demonstrate that..." etc.
- "studies witnessed..."
- "grounded on the JD-R model"
- "however there is lag of studies that insight the moderating role..." both lag and insight are problematic in this sentence
- "has been witnessed in the literature"
- Generally, other terms/phrases to consider could include: "evidence suggests that..." "has been demonstrated by X studies" "has been shown to X" 
- "The theory argues that doctoral student appraise the program demands depending on their subjective environment as challenge or hinderance demands" - this is confusing and needs to be reworded - perhaps making doctoral student[s] plural, perhaps adding on how they view their subjective environment, perhaps adding "the" ahead of the word challenge - or perhaps changing to "doctoral student appraisal of program demands depends on...demands change"?
- "in this connection, one may concur..." - surmise, perhaps? (in this context "connection" does not make sense)
- "....(COR), we explain the doctoral program is bound with resources along with the demands" - unclear - maybe add "explain that..." and "bound" does not make sense - unsure what word is intended
- "...the program journey situate..." - replace situate
- "foster the study engagement..." - replace foster
- "based on SDT articulation..." - replace articulate
- "despite the 11 studies borrowed from the SDT to understand the significance of intrinsic motivation, there are only two studies have witnessed..." - "borrowed" does not make sense, "witness" does not make sense, there are/have witnessed grammar/tense do not match... revise sentence
- Proposition 3 needs to be reworded - "higher autonomy" does not make sense - "greater autonomy," perhaps? - emphasizes does not make sense - "amplifies" or "increases," perhaps? - "a greater level of ..." does not make sense, "more autonomous feelings..." or "greater perception of self-determination by graduate students..." perhaps? - "foster" in the current context should be plural - and both sentences need to be revised, currently its guesswork to try to figure out what the authors mean here.

3. There are a number of consistent grammatical errors, such as errors in plural versus singular nouns/verbs and missing or incorrect prepositions. These make it very hard to follow the flow and meaning of sentences in some cases and need to be revised. Examples are included below, but the manuscript should be checked carefully for grammar, sentence structure, and wording choice throughout.

- Abstract - "theoretical underpinnings of demands-resources and mental well-being are reported" - is there a word missing here - do the authors mean the demands-resources framework/model?
- similarly, "jobs demands and resources" throughout - add model/framework or rework the sentence to use the singular form if generally referring to the construct and not the model
- "viz" - unsure if the authors mean "vis-à-vis," perhaps?
- "resulted in unpleasant experience in..." - unpleasant does not make sense in this context
- "these demands are conceptualized as 'two-factor stress model' or 'differentiated demands model'" - unclear if words are missing, perhaps "the" ahead of each model - or perhaps "as part of" - or maybe just awkward wording?
- "lack of infrastructures" should be singular not plural
- "unlike other resources, the intrinsic motivation is a significant..." - "the" should be removed along with "significant" (see earlier comment above)
- "in turn, improves the health of the doctoral students..." should remove second "the"
- "through the job strain as mediator" - remove "the"
- "Greater complexity and responsibility in the doctoral program foster the study engagement of the doctoral students that internally lessens the burnout" - unclear what is meant by "the study engagement" - do the authors mean engagement is improved by autonomous motivation to engage in the students' own research? Clarify/rephrase
- "Thus future research can explore how the autonomous orientation motivates...." - remove "the"
- "There are limited studies that examined the effect of demands-resources on wellbeing..." grammatically confusing, revise - "have examined the interaction of demand and resources," perhaps? - or "have examined the demands-resources model in the context of..."?
- "Researchers are mandated with a high degree of autonomy..." - "mandated" does not make sense, reword - "tend to display a high degree," perhaps?
- "The proposed framework... serves as an implication...by incorporating challenge-demands in their doctoral education" - has implications perhaps? - incorporating the challenge-demands framework perhaps? - very confusing sentence as currently written.
- "Institutions are suggested to frame..." - different word needed - "Recommendations for institutions include reframing the workload..." perhaps?

Other wording suggestions: 

 "Scope for future" more commonly referred to as "Future Directions" as the header title

Are the rationale for, and objectives of, the Systematic Review clearly stated?

Yes

Is the statistical analysis and its interpretation appropriate?

Not applicable

Are sufficient details of the methods and analysis provided to allow replication by others?

Yes

Are the conclusions drawn adequately supported by the results presented in the review?

Partly

Reviewer Expertise:

Biomedical graduate education training and education; training environment, professional development resources/accessibility, and mental health and well-being in graduate populations; intersectional approached to equitable environments in academic and research culture; doctoral and postdoctoral (PhD) career development

I confirm that I have read this submission and believe that I have an appropriate level of expertise to state that I do not consider it to be of an acceptable scientific standard, for reasons outlined above.

# Response

**Published:** 2023-07-21

**Authors:** Vrinda Acharya

**Affiliations:** Manipal University, India

Title of the article "A Framework for doctoral education in developing students’ mental well-being by Integrating the Demands and Resources of the Program: An integrative review"

 Appreciate your constructive feedback and detailed report on the article's improvement. The author team will definitely work on your suggestions and resend the revised article at the earliest. We will work rigorously on grammar, sentence structure, and wording choice of the article.

 Thank you once again for the recommendations and suggestions.

 Regards

 Vrinda
